# Development of Transgenic Cloned Pig Models of Skin Inflammation by DNA Transposon-Directed Ectopic Expression of Human β1 and α2 Integrin

**DOI:** 10.1371/journal.pone.0036658

**Published:** 2012-05-10

**Authors:** Nicklas Heine Staunstrup, Johannes Madsen, Maria Nascimento Primo, Juan Li, Ying Liu, Peter M. Kragh, Rong Li, Mette Schmidt, Stig Purup, Frederik Dagnæs-Hansen, Lars Svensson, Thomas K. Petersen, Henrik Callesen, Lars Bolund, Jacob Giehm Mikkelsen

**Affiliations:** 1 Department of Biomedicine, Aarhus University, Aarhus, Denmark; 2 Department of Disease Pharmacology, LEO Pharma, Ballerup, Denmark; 3 Department of Animal Science, Aarhus University, Tjele, Denmark; 4 College of Animal Science and Technology, Nanjing Agricultural University, Nanjing, China; 5 Department of Veterinary Reproduction and Obstetrics, University of Copenhagen, Frederiksberg, Denmark; 6 HuaDa JiYin (BGI), Shenzhen, China; University of Connecticut, United States of America

## Abstract

Integrins constitute a superfamily of transmembrane signaling receptors that play pivotal roles in cutaneous homeostasis by modulating cell growth and differentiation as well as inflammatory responses in the skin. Subrabasal expression of integrins α2 and/or β1 entails hyperproliferation and aberrant differentiation of keratinocytes and leads to dermal and epidermal influx of activated T-cells. The anatomical and physiological similarities between porcine and human skin make the pig a suitable model for human skin diseases. In efforts to generate a porcine model of cutaneous inflammation, we employed the Sleeping Beauty DNA transposon system for production of transgenic cloned Göttingen minipigs expressing human β1 or α2 integrin under the control of a promoter specific for subrabasal keratinocytes. Using pools of transgenic donor fibroblasts, cloning by somatic cell nuclear transfer was utilized to produce reconstructed embryos that were subsequently transferred to surrogate sows. The resulting pigs were all transgenic and harbored from one to six transgene integrants. Molecular analyses on skin biopsies and cultured keratinocytes showed ectopic expression of the human integrins and localization within the keratinocyte plasma membrane. Markers of perturbed skin homeostasis, including activation of the MAPK pathway, increased expression of the pro-inflammatory cytokine IL-1α, and enhanced expression of the transcription factor c-Fos, were identified in keratinocytes from β1 and α2 integrin-transgenic minipigs, suggesting the induction of a chronic inflammatory phenotype in the skin. Notably, cellular dysregulation obtained by overexpression of either β1 or α2 integrin occurred through different cellular signaling pathways. Our findings mark the creation of the first cloned pig models with molecular markers of skin inflammation. Despite the absence of an overt psoriatic phenotype, these animals may possess increased susceptibility to severe skin damage-induced inflammation and should be of great potential in studies aiming at the development and refinement of topical therapies for cutaneous inflammation including psoriasis.

## Introduction

Development and refinement of topically administered medications directed against cutaneous inflammatory and hyperproliferative conditions, like psoriasis, scleroderma, and cutaneous lupus erythematosus, demand suitable animal models with a skin architecture that mimics the human counterpart. The skin of pigs greatly resembles that of humans with respect to morphology and physiology [1]. The thickness of the epidermis of pig and human skin is comparable, and turnover rate of epidermal cells as well as uptake of topically applied drugs are similar [2].

Integrins play a central role in cell-to-cell and cell-to-extracellular matrix interactions involved in inside-out and outside-in signaling, contributing to the control of cell function and behavior [3], [4]. Integrins constitute a superfamily of αβ-heterodimer transmembrane receptors that recognize predominately extracellular matrix (ECM) ligands and convey outside-in signaling events such as cytoplasmic alkalization, potassium channel activation, and activation of various signaling molecules, including mitogen-activated protein kinases (MAPKs) and protein kinase C (PKC). This entails great importance for many cellular aspects such as proliferation, differentiation, and migration [3], [5]. Epidermal keratinocytes express several extracellular matrix receptors including α2β1, α3β1, and α5β1 that bind collagen, laminin, and fibronectin, respectively. In keratinocytes, β1 integrins regulate stratification and terminal differentiation and are regarded as a stem cell marker of the epidermis [6]. Besides signals exclusively mediated by integrins, integrin-directed signal transduction may involve synergistical cross-talk with other membrane proteins such as caveolin [7], the vascular endothelial growth factor receptor (VEGFR) [8] and epidermal-growth-factor receptor (EGFR) [9], [10]. Integrin β1 can induce EGF receptor and platelet derived growth factor (PDGF) β-receptor tyrosine phosphorylation even in the absence of receptor ligands, which subsequently leads to phosphorylation of the Shc adaptor protein and MAPK activation [9], [11], [12]. In epithelial cells, β1 integrins localize at cell-to-cell boundaries [13], leading to the activation by phosphorylation of factors such as EGFR, suggesting, hence, a role for β1 integrin in the direct interaction between cells [11].

Subrabasal overexpression of α2 and/or β1 integrin (encoded by the human ITGA2 and ITGB1 genes, respectively) has previously been shown to entail dysregulation of epidermal proliferation and differentiation in mice, leading to cutaneous influx of activated cells of the leukocyte lineage and development of a psoriasis-like phenotype [14]. In non-lesional human skin, expression of the ITGB1 gene is confined to keratinocytes in the epidermal basal layer (stratum basale) via complex transcriptional and posttranslational regulatory mechanisms [15], [16]. Stem cells within the basal layer self-renew but give rise also to non-stem daughter cells that detach from the basement membrane and transit into the subrabasal layers. These transient amplifying cells undergo a limited number of cell-divisions before they become committed to terminal differentiation. However, in wound healing and in several cutaneous diseases such as psoriasis and lichen planus, ITGB1 expression is maintained in keratinocytes of the subrabasal layer [17], causing these cells to remain proliferative and to disturb terminal differentiation. Loss of keratinocyte-ECM adhesion leads to an abrupt arrest in proliferation and induces differentiation. Suspended keratinocytes are in contrast prevented from differentiation by treatment with antibodies to β1 integrin, thus emphasizing the necessity of integrin expression for cell division and negative regulation of differentiation [18], [19]. Importantly, the abnormal expression pattern of β1 integrin in lesional psoriatic skin is not a secondary effect of cutaneous inflammation, since intradermal injections of cytokines do not induce suprabasal β1 integrin expression [20]. Hence, this pattern is caused, at least partially, by gene regulatory abnormalities.

Creation of transgenic animals by somatic cell nuclear transfer (SCNT) has been described for a variety of animals including sheep [21], mouse [22], [23], cow [24], [25] and pig [26], [27], [28]. Recently a simplified handmade cloning (HMC) procedure has been devised [29], [30], which has been applied for the generation of genetically engineered pigs [31], including Göttingen minipigs with constitutive and ubiquitous expression of eGFP [32] and minipigs expressing the APP gene containing a dominant mutation associated with early onset of Alzheimer's disease [33].

Stable genetic manipulation of somatic donor cells can be achieved through various mechanisms including random genomic insertion of plasmid DNA [34], [35] and transduction with recombinant retroviral vectors derived from gamma-retroviruses or lentiviruses [36], [37]. Recently, we described a novel approach employing Sleeping Beauty (SB) DNA transposon-based vectors to generate genetically engineered Göttingen minipigs [32]. SB, a Tc1/mariner-like transposable element reconstructed from teleost fish [38], exhibits ‘cut-and-paste’ transposition in a variety of vertebrate species with accurate and near-random genomic insertion of the genetic cargo of interest [39], [40]. For this reason, SB has been developed as a gene-inserting vector for both therapeutic applications [41], [42], [43] and transgenesis [44], [45], [46].

In this report, we utilize SB DNA transposition and HMC for development of pig models of chronic skin inflammation. We describe the generation of transgenic Göttingen minipigs with subrabasal expression of human integrins β1 and α2 driven by the involucrin promoter. All transgenic pigs demonstrate molecular hallmarks of skin inflammation including activation of the MAPK pathway, upregulation of interleukin-1α (IL-1α), and enhanced expression of c-Fos. Notably, we find that cellular dysregulation obtained by overexpression of either β1 or α2 integrin occurred through different cellular signaling pathways. These findings mark the creation of the first cloned pig models with molecular markers of skin inflammation.

## Results

### Establishment of persistent β1 integrin expression by SB transposition

**Figure 1:**
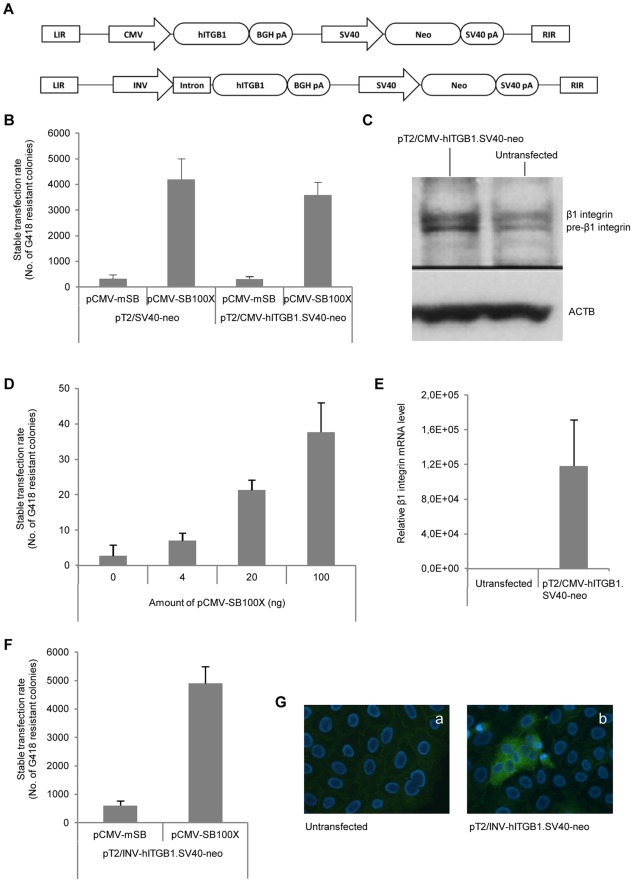
Functional analysis of bicistronic SB transposon vectors. (**A**) Schematic representation of bicistronic SB-transposon vectors carrying the hITGB1 gene driven by CMV and INV promoters, respectively. LIR and RIR indicate the SB transposon left and right inverted repeats, respectively; SV40 pA and BGH pA, indicate the Simian virus 40 and bovine growth hormone polyadenylation sites, respectively. (**B**) Stable transfection rate in NIH3T3 cells co-transfected with equimolar amounts of pT2/SV40-neo or pT2/CMV-hITGB1. SV40-neo in conjunction with pCMV-mSB or pCMV-SB100X. pUC19 was included as stuffer in some transfections to ensure that equal amounts of DNA were transfected. G418-resistant colonies were counted after 14 days selection. (**C**) Western blot analysis using a mouse anti-human β1 integrin mAb (610467) on cellular protein extracts from naïve NIH3T3 cells or NIH3T3 cells stably transfected with pT2/CMV-hITGB1. SV40-neo (top panel). Top band is mature β1 integrin, lower band pre-β1 integrin. Detection of ACTB with an anti-β-actin mAb served as loading control (lower panel). (**D**) Transposase titration assay in Göttingen primary fibroblasts co-transfected with 0–100 ng pCMV-SB100X and 1.9 µg pT2/CMV-hITGB1. SV40-neo. Resistant colonies were counted after 14 days of selection with G418. (**E**) Quantitative RT-PCR on total mRNA extracted from naïve or pT2/CMV-hITGB1. SV40-neo stably transfected Göttingen primary fibroblasts. Human β1 integrin specific exon-exon primers were utilized to detect transcripts derived from the integrated transposon. The mRNA level was normalized to the level of endogenous β-actin mRNA. (**F**) Transposition assay in HaCaT cells co-transfected with 1.9 µg pT2/SV40-neo or pT2/INV-hITGB1. SV40-neo in conjunction with 100 ng pCMV-mSB or pCMV-SB100X. Resistant colonies were counted following 14 days of G418 selection. (**G**) Immunostaining of permeabilized naïve (a) or pT2/INV-hITGB1. SV40-neo-transfected (b) HaCaT cells. A mouse anti-human β1 integrin mAb (P5D2) was used to identify β1 integrin protein derived from the transposon expression cassette. Data are presented as mean values ± standard deviations.

We initially generated four bicistronic SB vectors in which an eGFP expression cassette (CMV-eGFP) was inserted in forward or reverse orientation either upstream or downstream of a neomycin resistance cassette (SV40-neo) within the context of an optimized T2 SB transposon [47]. Variants with the eGFP cassette situated upstream of SV40-neo were most efficiently transposed and displayed stable expression for more than six weeks (data not shown). A vector design with a forward orientation of both expression cassettes was utilized in further studies and for construction of a transposable vector carrying the hITGB1 gene driven by a CMV promoter (**Fig. 1A**). We utilized the ITGB1_A_ isoform, as this variant is ubiquitously expressed and is known to localize at focal adhesion contacts where it interacts with intracellular factors.

To express the human β1 integrin in cells that did not already express human integrin, we stably transfected NIH3T3 murine fibroblasts with the T2/CMV-hITGB1. SV40-neo transposon. Cells were transfected with either pT2/SV40-neo or pT2/CMV-hITGB1. SV40-neo together with either pCMV-SB100X or pCMV-mSB, expressing the hyperactive SB100X transposase and the catalytically inactive mSB transposase, respectively. Comparable levels of transposition were measured for the two transposons, as indicated by the amount of G418-resistant NIH3T3 colonies appearing after selection (ranging from 3.600 ± 400 colonies for T2/CMV-hITGB1. SV40-neo to 4.200 ± 700 colonies for T2/SV40-neo; **Fig. 1B**), indicating that the size difference of 4.5 kb between the two vectors did not significantly affect the rate of transposition. Under the experimental conditions used, the stable transfection rate for pT2/CMV-hITB1. SV40-neo was increased 12-fold in the presence of SB100X relative to the control (**Fig. 1B**).

Western blot analyses carried out with antibodies recognizing both human and mouse β1 integrin showed increased band intensity in pools of stably transfected NIH3T3 cells relative to naïve NIH3T3 cells (**Fig. 1C**). In many cell types, including murine fibroblasts, ITGB1-encoded protein is synthesized in excess as a pre-β1-integrin confined to the endoplasmic reticulum. The maturation of pre-β1-integrin through post-translational modifications results in an increase in the molecular weight to 130 kDa. Elevated levels of both pre-β1-integrin (lower band) and maturated β1-integrin (higher band) were detected, suggesting that human β1 integrin was properly processed at the post-translational level.

To determine the optimal transposase:transposon plasmid ratio for SB transposition, we co-transfected Göttingen primary fibroblasts with 1.9 µg pT2/CMV-hITGB1.SV40-neo and varying amounts of SB100X-encoding plasmid (from 0 to 100 ng) and counted G418-resistant colonies. Although the efficiency was markedly lower than in NIH3T3 cells, an increased stable transfection rate was measured with increasing amounts of pCMV-SB100X, resulting at most in 38±8 colonies per transfection (**Fig. 1D**). To verify expression of the hITGB1 transgene in G418-resistant Göttingen primary fibroblasts, we performed qRT-PCR specific for the human ITGB1 transcript on total RNA derived from pools of stably transfected cells. Whereas the transcript was undetectable in untransfected fibroblasts, a solid increase in the level of mRNA encoding the β1 integrin could be measured in cells carrying the transposon vector (**Fig. 1E**). In summary, our findings demonstrated that stable transfection mediated by SB transposition facilitated persistent expression of human β1 integrin in primary porcine cells. To reduce the risk of inserting the SB100X-encoding plasmid into genomic DNA by random insertion, we chose to use 4 or 20 ng of pCMV-SB100X for further studies in Göttingen primary fibroblasts.

### Construction of transgenic blastocysts for pig cloning

To obtain transgenic animals with transgene expression confined to the suprabasal layer of the skin, we substituted the CMV promoter of pT2/CMV-hITGB11V40-neo with the suprabasal keratinocyte-specific involucrin (INV) promoter (see **Fig. 1A** for schematic representation of vector). Downstream of the promoter, we included the first intron of the involucrin gene, as this has been shown to elevate expression [48]. By interchanging the CMV promoter for the INV promoter, the size of the transposon was enlarged from 6.8 kb to 10.1 kb. However, evaluation of transposition efficiency by colony formation assays in the human keratinocyte cell line HaCaT showed potent levels of insertion in the presence of SB100X compared to the background level observed in the presence of the mSB transposase (**Fig. 1F**). Moreover, immunostaining with an antibody specific for human β1 integrin demonstrated an expected staining of the membrane-bound endogenous β1 integrin as well as a pancellular distribution of β1 integrin in cells carrying the T2/INV-hITGB1.SV40-neo transposon (**Fig. 1G**).

Next, we introduced the INV-driven transgene cassette in Göttingen primary fibroblasts to produce donor cells for pig transgenesis by HMC. Co-transfection of the SB vector with pCMV-SB100X resulted in an insertion rate that was 12-fold higher than the background level (data not shown). More than 100 G418-resistant colonies were pooled and subsequently utilized as donor cells in the HMC procedure. The blastocyst rate was 47.8%, and a total of 357 reconstituted embryos were surgically transferred to a total of three recipient sows. All sows became pregnant and gave rise to a total of 23 piglets after induction of labor with prostaglandin 24 h before term. None of the pigs had visible gross abnormalities apart from one with several malformations as cleft palate, syndactyly, polydactyly and kyphosis. Nevertheless, only 6 piglets survived the critical first two weeks after birth. Autopsy of the dead piglets showed agenesis of the gall bladder in most of them. An overview is provided in **Supplementary** **Table S1**.

### Demonstration of transgenic status for all six piglets

**Figure 2:**
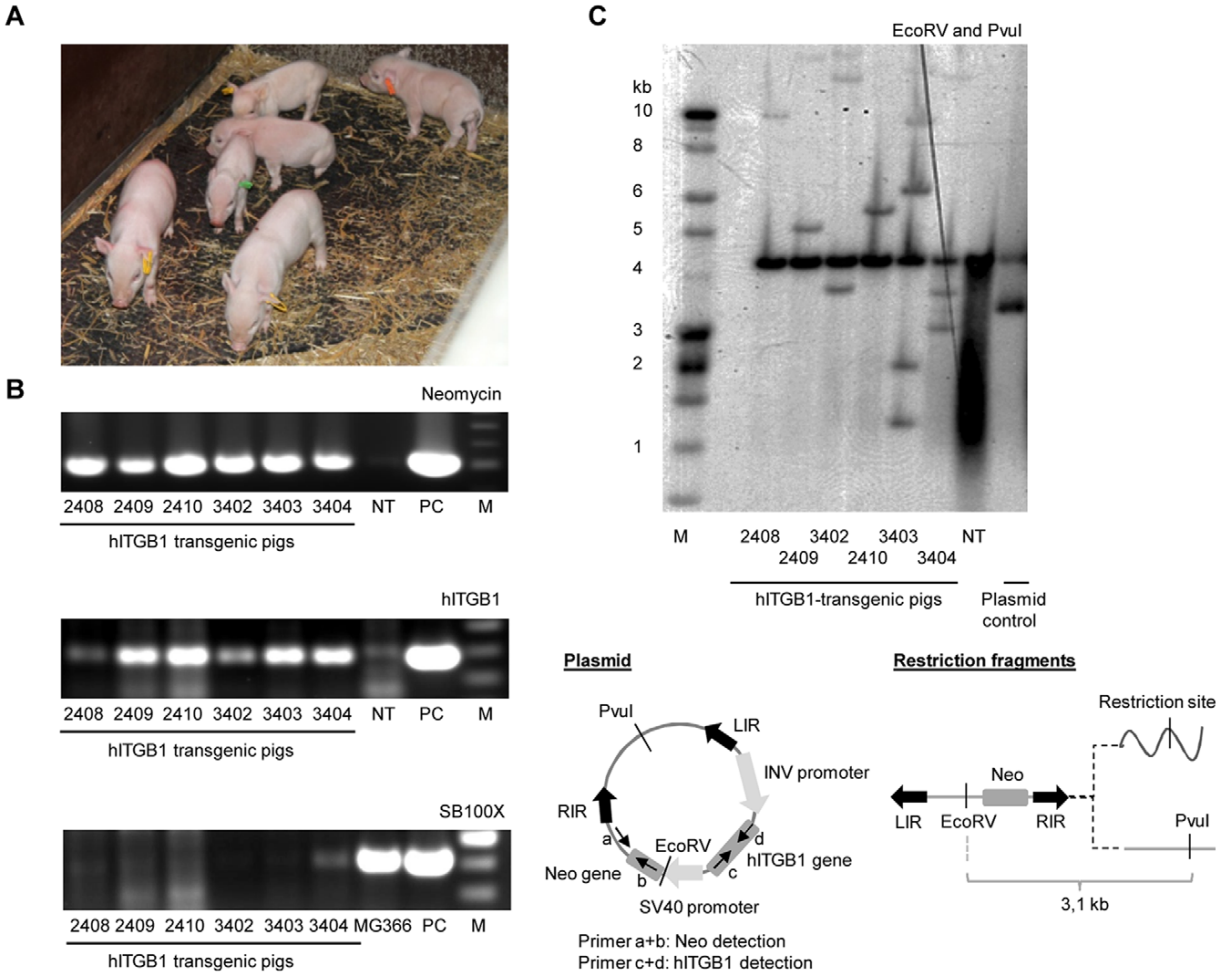
Demonstration of transgenesis and lack of SB100X gene integrations by genotyping of cloned pigs produced by HMC. (**A**) Photo of the six cloned pigs at one week of age. (**B**) PCR analysis on genomic DNA extracted from cultured pig fibroblasts. Neomycin- and hITGB1-specific internal primer-pair demonstrated the presence of the transgenic cassette in the cloned pigs but not in the non-transgenic control (NT) (two upper panels). Random integration of the SB100X gene could not be detected in the cloned pigs by using SB100X-specific primers (lower panel). MG366, a cloned pig with a known SB100X insertion was used as a control; PC, plasmid control; M, 100 bp marker. (**C**) Southern blot analyses on 15 µg genomic DNA from the transgenic animals demonstrated between one and six insertions per pig. DNA was digested with EcoRV and PvuI (left panel) or with NheI and SalI (data not shown) and probed with a ^32^P-labelled Neo probe. DNA from NT spiked with pT2/INV-hITGB1.SV40-neo was utilized as plasmid control. The number of insertions ranges from one to five in the six transgenic animals. An identical band appears in all lanes and is presumably non-specific. NT, non-transgenic control.

The surviving piglets (**Fig. 2A**) were housed together and gained weight according to the normal growth curve of the herd. Fibroblasts were grown from ear biopsies taken from the six piglets at the age of 2 weeks and from a non-transgenic age-related animal. PCRs on fibroblast-derived genomic DNA (using primers specific for the neo gene and the hITGB1 gene, respectively) produced fragments of the expected size for each cloned piglet with no visible bands from the non-transgenic control pig (**Fig. 2B**, upper two panels), suggesting that all animals were indeed transgenic. A PCR specific for the SB100X gene was negative for all pigs, demonstrating that passive integration of the SB100X-encoding plasmid had not occurred (**Fig. 2B**, lower panel). In addition, fibroblasts from three stillborn littermates also proved transgenic (data not shown). Thus, nine out of nine analyzed animals were indeed positive for the human β1 integrin.

To determine the SB vector copy number in each animal, we performed Southern blot analysis on genomic DNA isolated from blood samples using two different sets of restriction enzymes. The analysis showed two pigs with each one insertion, two pigs with three insertions, and two pigs carrying each two and five insertions (**Fig. 2C** and data not shown). Notably, all pigs were genetically distinct and therefore arose from different donor clones, although they were generated from the same pool of donor cells. Also, the band pattern suggested that three of the pigs (all with >1 insertion) contained an integrant generated by random insertion of the transposon plasmid (data not shown). Karyotyping of all six pigs, showed a normal diploid karyotype without any visible chromosomal abnormalities in all cases (data from one representative pig are shown in **Supplementary** **Fig. S1**). A total of five distinct insertion sites from the six pigs were identified, of which two could be matched to the Sscrofa9.56 porcine genome database (Ensembl). Four of the sites featured the TA-dinucleotide flanking the transposon sequence, a hallmark of transposase-directed insertion, whereas one event of random integration was identified. A summary of the genetic status of each of the six pigs is provided in **Supplementary** **Table S2**.

### Evidence of transgene expression in transgenic primary keratinocytes

**Figure 3:**
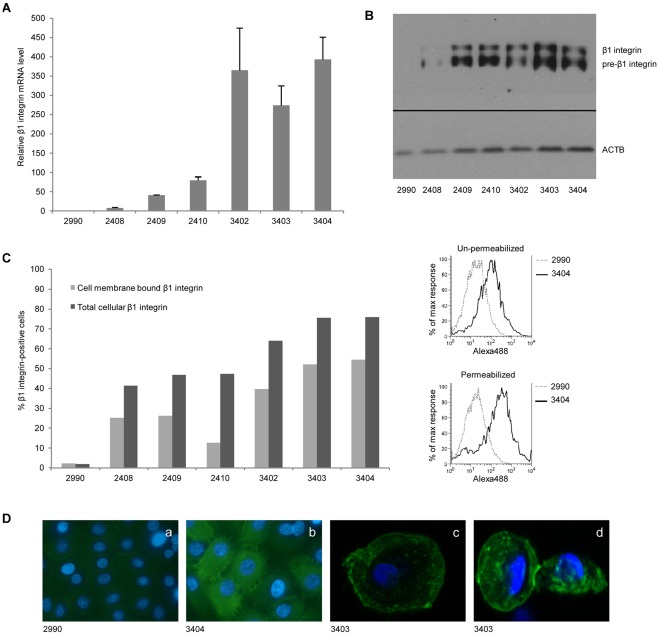
Expression and cell membrane localization of heterologous β1 integrin. (**A**) Quantitative RT-PCR for quantification of β1 integrin mRNA performed on total mRNA extracted from cultured keratinocytes derived from a non-transgenic pig (#2990) and the six hITGB1 transgenic pigs, normalized to endogenous β-actin. The expression level of human β1 integrin was proportional to genomic integration copies and only detectable in the transgenic animals. Data are presented as mean values ± standard deviations. (**B**) Western blot analysis on total cellular protein from the keratinocytes described in (A). Employing the mouse anti-human β1 integrin mAb (610467), both pre- and post-translationally processed human β1 integrin protein is detected in all transgenic pigs. ACTB detection was used as loading control. (**C**) Flow cytometry analysis of keratinocytes stained with an anti-human β1 integrin mAb (P5D2) without permeabilization (light gray bars) or with saponin permeabilization (dark gray bars). Representative histograms comparing fluorescence from pig #2990 and #3404 with and without permeabilization are depicted. In five out of the six hITGB1 transgenic keratinocytes, β1 integrin was predominately localized to the cell membrane. (**D**) Representative images from wide-field (a–b) and confocal (c–d) fluorescence microscopy of permeabilized keratinocytes (derived from the non-transgenic control (#2990) and hITGB1 transgenic pigs) stained with anti-human β1 integrin (P5D2). Detection of β1 integrin was restricted to transgenic cells with accumulation at the plasma membrane and especially at cell-cell adhesion sites.

Transgene expression was assessed in *in vitro*-cultured keratinocytes that were allowed to expand from skin explants from each of the transgenic pigs as well as from a non-transgenic control pig (pig #2990). The keratinocytes were cultured in the presence of EGF, which is known to stimulate dimerization and activation of integrin αβ1 complexes. Firstly, solid expression of the hITGB1 gene was verified by qRT-PCR using exon-exon primers specific for hITGB1 and porcine ACTB primers for internal control (**Fig. 3A**). Notably, the highest expression level was detected in animals with 3, 5, and 2 insertions (pigs #3402, #3403, and #3404, respectively), suggesting a correlation between the copy number and the expression level. Secondly, Western blot analysis using an antibody that does not show cross-reactivity with porcine β1 integrin, demonstrated high levels of hITGB1-encoded protein in all transgenic pigs with the exception of pig #2408 (**Fig. 3B**), which was in correlation with the RNA expression data. The detection of both pre-β1-integrin and matured β1 integrin indicated that the protein was correctly processed after translation in transgenic porcine keratinocytes. Integrin dimerization and membrane incorporation is a prerequisite for integrin-dependent signal transduction. Hence, thirdly, we used flow cytometry to evaluate to which extent subunits of integrin were anchored in the membrane of the keratinocytes. Subpopulations of keratinocytes from each pig were stained with antibodies specific for β1 integrin and keratin 14 (K14) following no or mild saponin permeabilization. Although this β1 antibody displays some porcine cross-reactivity, the percentage of keratinocytes expressing β1 integrin was found to be markedly increased (at least 20-fold) in all transgenic animals relative to the non-transgenic pig (**Fig. 3C**). Equally important, in most transgenic pigs a larger proportion (approximately 60%) of the β1 integrin that could be detected by flow analysis was embedded in the membrane of keratinocytes (**Fig. 3C**).

Finally, fluorescence microscopy after anti-integrin β1 immunostaining demonstrated pancellular expression of β1 integrin in keratinocytes from transgenic pigs, as opposed to cells from the control animal (pig #2990) that were negative for expression (**Fig. 3D**, panels a and b). Notably, differentiating cells produced a brighter signal, which is in concordance with the activity of the involucrin promoter only in cells committed to differentiation. Confocal microscopy further demonstrated that human β1 integrin was anchored primarily in the cellular membrane and that this localization was reproducibly predominant at cell-to-cell contacts (**Fig. 3D**, panels c and d). This supported the overall notion that the integrin proteins were properly processed, dimerized, and distributed in keratinocytes of the transgenic pigs, rendering them likely to interact with endogenously expressed membrane receptors.

### Subrabasal expression of hITGB1-encoded protein

**Figure 4:**
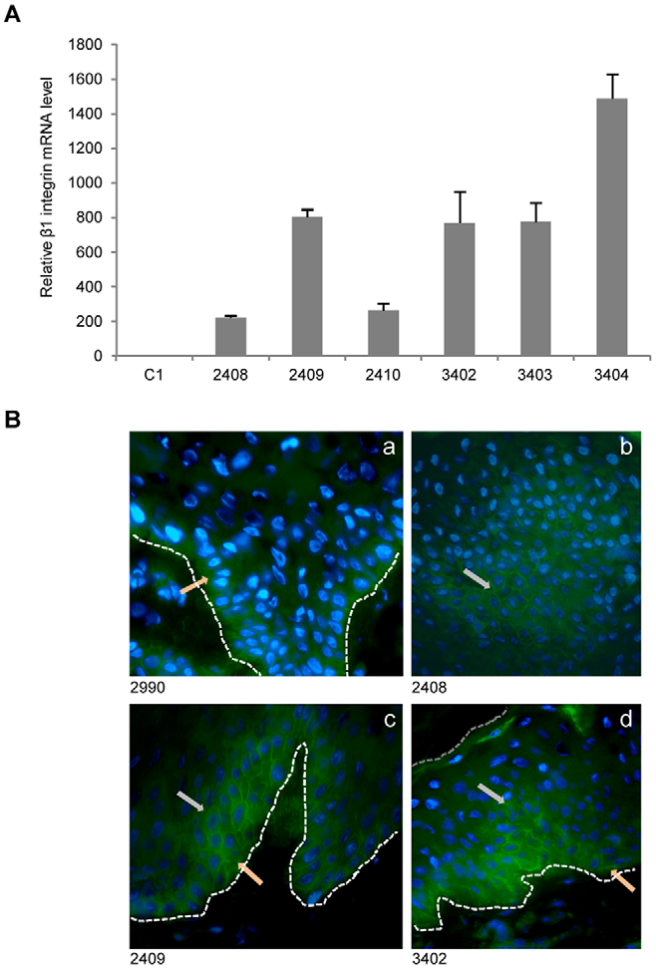
Ectopic expression of the β1 integrin in the transgenic pigs. (**A**) Total RNA obtained from non-transgenic (#C1) and hITGB1 transgenic pig skin-biopsies were analyzed by human β1 integrin specific qRT-PCR, normalized to endogenous β-actin. Significant expression was detected in all transgenic pigs. Data are presented as mean values ± standard deviations. (**B**) Representative wide-field fluorescence microscopy images of permeabilized frozen cutaneous sections (from a non-transgenic pig (#2990) and hITGB1-transgenic pigs) stained with an anti-human β1 integrin mAb (P5D2). Staining of basal cells is evident in all pigs (peach arrows), but subrabasal expression is apparent in transgenic animals only (grey arrows). Basal membrane is indicated by a white dashed line and the skin surface by a grey dashed line. In b the basal membrane is located to the lower right outside the figure.

**Figure 5:**
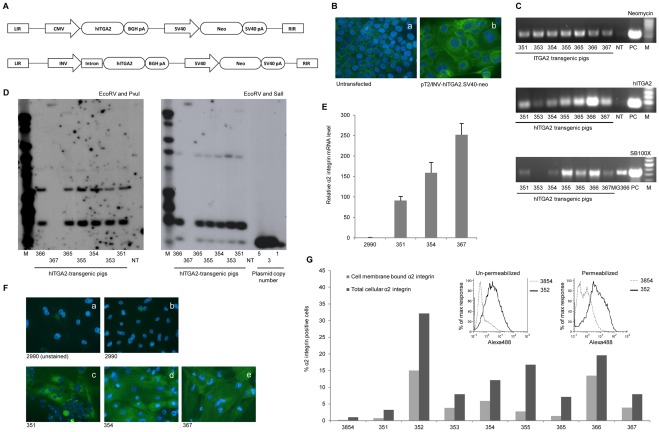
Generation of hITGA2-transgenic pigs with ectopic expression of human α2 integrin. (**A**) Schematic figure of bicistronic SB transposon vectors with expression of human α2 integrin driven by the CMV and INV promoters and the neo selection marker gene driven by the SV40 promoter. LIR and RIR indicate SB transposon left and right inverted repeats, respectively; SV40 pA and BGH pA indicate Simian virus 40 and bovine growth hormone polyadenylation sites, respectively. (**B**) Immunostaining of permeabilized naïve (a) or pT2/INV-hITGA2.SV40-neo-transfected (b) HaCaT cells with a mouse anti-human α2 integrin mAb (P1E6). Background staining of endogenous α2 integrin is evident in naïve cells, whereas transfected cells show intense pancellular staining. (**C**) PCR analysis on genomic DNA extracted from cultured hITGA2-transgenic pig fibroblasts. The presence of Neo and hITGA2 was confirmed for all transgenic pigs with specific primer-pairs (upper panels). Passive integration of SB100X could be verified in all the cloned pigs by the use of primers specific for the SB100X sequence (lower panel). NT, non-transgenic control; MG366, cloned pig with known insertion of the SB100X gene; PC, plasmid control; M, 100 bp marker. (**D**) Southern blot analyses performed with 11 µg genomic DNA extracted from hITGA2-transgenic pig fibroblasts showed that all pigs, except 367, were genetically identical. DNA was digested with EcoRV and PvuI (left panel) or with EcoRV and SalI (right panel) and probed with a ^32^P-labelled Neo probe. DNA from NT spiked with pT2/INV-hITGA2.SV40-neo was utilized in copy number controls with 1, 5 and 10 copies, respectively (refer to figure 2C for lengths of restriction fragments). NT, non-transgenic control; M, 1 kb marker. (**E**) qRT-PCR analysis of α2 integrin mRNA expression levels in hITGA2-transgenic pig keratinocytes, normalized to endogenous β-actin. Expression of human α2 integrin mRNA was evident in all transgenic pig keratinocytes. Representative data are presented as mean values ± standard deviations. (**F**) Representative wide-field fluorescence microscopy images of permeabilized and unstained or immunostained (with anti-human α2 integrin mAb (P1E6)) frozen cutaneous sections from the non-transgenic pig #2990 (a–b) and analyzed hITGA2-transgenic pigs (c–e). A clear staining for human α2 integrin protein was present only in transgenic keratinocytes. (**G**) Flow cytometry analysis on hITGA2-transgenic keratinocytes stained with an anti-human α2 integrin mAb (P1E6) without permeabilization (light gray bars) or with saponin permeabilization (dark gray bars). Also shown are representative histograms comparing fluorescence intensities from pig 3854 and 352 with and without permeabilization.

To investigate the production of human β1 integrin in the skin of transgenic pigs, we performed qRT-PCR on total RNA isolated from skin biopsies derived from the six transgenic animals and a non-transgenic control pig (#C1). Robust levels of INV-driven expression were measured in all transgenic animals (**Fig. 4A**) with a variation between the pigs that was largely similar to the variation observed in the cultured keratinocytes. Immunostaining for β1 integrin on frozen skin sections revealed a clear staining of the epidermal basal layer for all pigs including the non-transgenic control animal (**Fig. 4B**) as a result of species cross-reactivity of the antibody. However, suprabasal intrusions of β1 integrin expression were visible only in sections from transgenic animals (**Fig. 5B**), indicating that the INV promoter actively promoted expression of the transgene in the suprabasal layer. Finally, histological sections of biopsies taken at age 1, 5, and 14 month(s) were stained with haematoxylin and eosin (**Supplementary** **Fig. S2**). There was no apparent difference in skin morphology between the transgenic pigs and the control pig, and we did not detect any morphological or phenotypic changes over a 14-month period.

### Production of transgenic pigs with ectopic expression of human α2 integrin

In parallel with the production of hITGB1-transgenic pigs, we initiated the development of pigs expressing human α2 integrin from the SB transposon construct pT2/INV-hIGTA2.SV40-neo (**Fig.** **5A**). Using pT2/INV-hIGTA2.SV40-neo, we established stable HaCaT keratinocyte clones and verified by immunostaining a solid expression of transgenic α2 integrin driven by the INV promoter. In contrast to naïve cells, an excessive amount of α2 integrin was distributed throughout the transgenic cells with a considerable proportion of the protein localized near the nucleus and a fraction residing in the plasma membrane (**Fig. 5B**). Göttingen primary fibroblasts were transfected with 1.9 µg pT2/INV-hITGA2.SV40-neo in conjunction with 4, 20, or 100 ng SB100X-encoding plasmid. Whereas transfection with 100 ng pCMV-SB100X produced the highest transposition ratio, 13-fold above background, cells transfected with 20 ng pCMV-SB100X were chosen for HMC in order to minimize the risk of spontaneously integrating the SB100X gene. About 30 clones were used in the HMC procedure, yielding a blastocyst rate of 43.6%. A total of 218 reconstructed embryos were equally distributed between two recipient sows, which after a successful pregnancy period delivered eight piglets (**Supplementary** **Table S3**). Unfortunately, six piglets were stillborn and another succumbed within the first two days after delivery. Autopsies of these animals showed agenesis of the gall bladder in all of them but did not unveil severe abnormalities that could be directly attributed as the cause of death.

Primary fibroblasts were grown from ear biopsies taken from all piglets except pig #352. The presence of the transgene-tagged transposon was verified in all pigs, including the live pig #351, by neo- and hITGA2-specific PCRs (**Fig. 5C**, upper two panels). Unexpectedly, we also detected the SB100X gene by PCR, indicating that the transposase helper plasmid had been accidentally inserted in the genome of these pigs. Notably, Southern blot analyses demonstrated that all pigs, except pig #367, were genetically identical, suggesting that they had originated from the same donor clone (**Supplementary** **Table S4**). This G418-resistant donor clone did not originate from an SB insertion event but rather from events of random plasmid insertion. Indeed, the Southern analysis suggested that the pigs carried two random plasmid insertions, one in which breakage of the plasmid had occurred outside the SV40-neo cassette and one in which breakage had occurred inside the neo gene (**Fig.5D**). The band pattern for pig #367 was compatible with a single transposase-directed insertion event.

Ectopic expression of the hITGA2 cassette was demonstrated by qRT-PCR and immunostaining for α2 integrin on total RNA and protein, respectively, from cultured transgenic keratinocytes derived from pigs #351, #354, and #367 (**Fig. 5E and 5F**). Notably, the levels of expression were found to differ between pigs that were genetically identical (comparing pigs #351 and #354), perhaps reflecting distinct epigenetic profiles in the two animals. Furthermore, pig #367 that harbored only a single integration demonstrated the highest level of expression. To estimate the extent to which transgenic α2 integrin was localized to the membrane, keratinocytes from the transgenic animals and a non-transgenic control (pig #3854) were either permeabilized with saponin or left untreated prior to staining with an anti-α2 integrin and an anti-K14 antibody. Flow cytometry analysis for expression of K14 and α2 integrin demonstrated robust anchoring of α2 integrin in the plasma membrane of transgenic keratinocytes, although the level of membrane-embedded α2 integrin was surprisingly heterogeneous (ranging from 16 to 69%) among the animals (**Fig. 5G**). To enlarge the herd of hITGA2-positive pigs, we performed an additional round of cloning using donor fibroblasts from hITGA2-transgenic animals, resulting in additional two live, transgenic piglets (#554 and #556) originating from a mixed pool of fibroblasts derived from pigs #355 and #366 (**Supplementary** **Table S5**).

The involucrin promoter should restrict the expression of hITGA2 to the subrabasal layers of the skin. To evaluate the expression level at different locations of the skin and in different tissues, we sacrificed pig #554, knowing that this pig might not be representative of the full herd due to its genetic make-up featuring random integrations. Samples from six distinct areas of the skin along with samples from eight internal organs including the liver, heart, and lung were obtained from pig #554. Quantitative RT-PCR on total RNA revealed comparable levels of human α2 integrin mRNA in all skin samples, whereas α2 integrin mRNA was not detectable in the control pig. Although considerable expression of α2 integrin mRNA was evident in all analyzed tissues of pig #554, expression of the transgene was significantly higher in all analyzed areas of the skin (p<0.0001) (**Supplementary** **Fig. S3**). These findings confirmed the tissue-specificity of the involucrin promoter, but also indicated that unsolicited expression from randomly integrated fragments was facilitated by endogenous promoter elements in pig #554.

### Activation of molecular markers of inflammation in the skin of pigs transgenic for either β1 or α2 integrin

A key feature of erroneous or engineered subrabasal expression of β1 integrin is the elevated expression and secretion of IL-1α which is not dependent on integrin ligation [49], [50]. The proinflammatory cytokine IL-1α plays pivotal auto- and paracrine roles in the inflammatory response inflicted in lesional psoriatic skin. Furthermore, the Erk/MAPK pathway is simultaneously activated by the autocrine effect of secreted IL-1α and by the integrin-dependent potentiating effect on EGFR transactivation [50].

**Figure 6:**
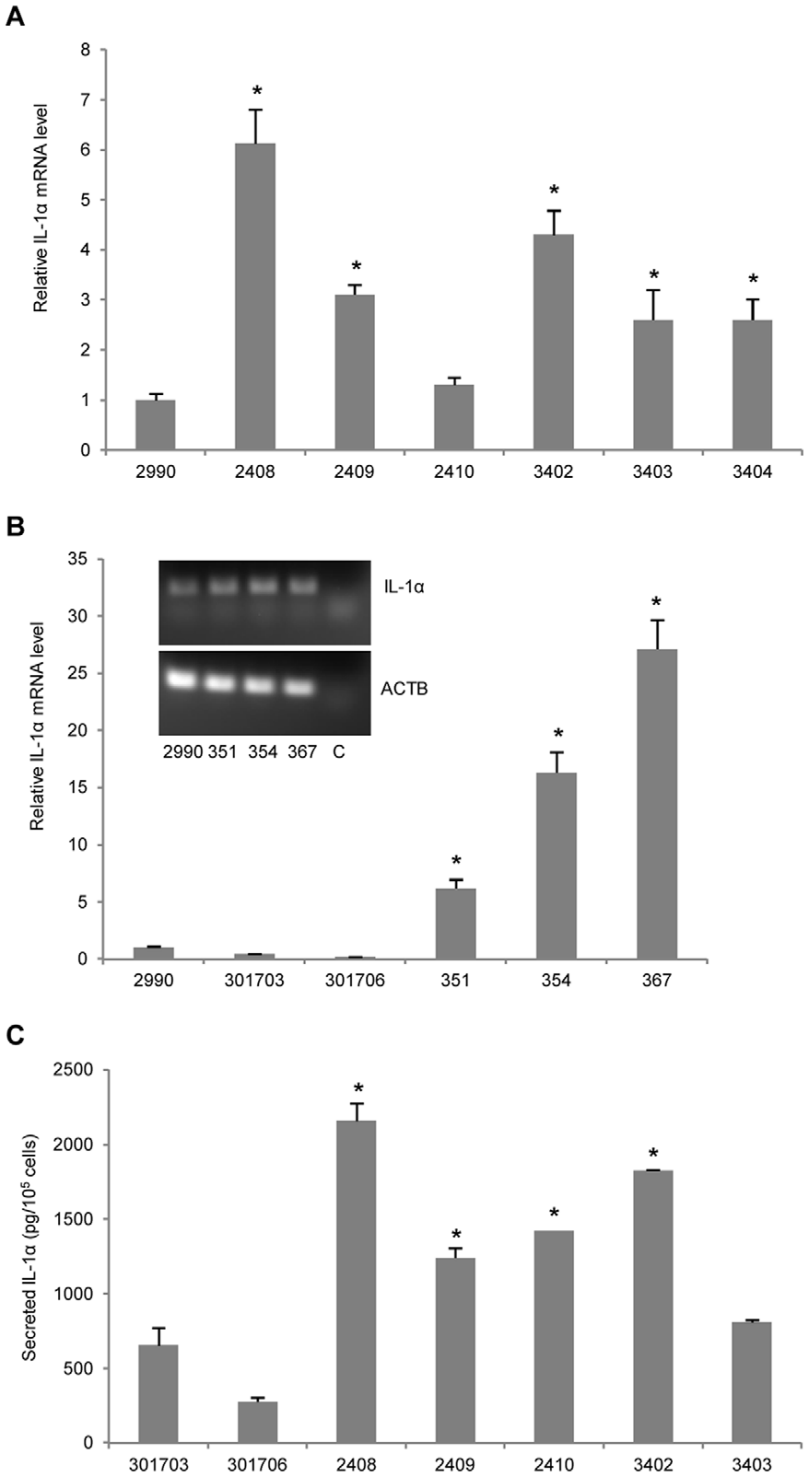
Increased production and secretion of IL-1α from transgenic keratinocytes. (**A**) Total RNA isolated from hITGB1-transgenic keratinocytes was used for porcine IL-1α specific qRT-PCR analysis. Expression levels of IL-1α were increased up to 6-fold in transgenic cells compared to a non-transgenic control (#2990). (**B**) qRT-PCR analysis for detection of IL-1α mRNA in hITGA2-transgenic keratinocytes. Messenger RNA levels were markedly elevated in transgenic cells compared to non-transgenic controls (#2990, #301703 and #301706). Images of gel-electrophoresed qRT-PCR products are depicted (insert); C indicates the water control. (**C**) Secretion of IL-1α measured by porcine IL-1α specific ELISA. Conditioned medium was harvested from hITGB1-transgenic keratinocytes and non-transgenic controls (#301703 and #301706) after 14 hours of incubation and used for ELISA. Secretion of IL-1α was increased up to 3-fold from transgenic cells. Data are presented as mean values ± standard deviations. Asterisks (*) indicate statistical significance compared to pig #2990 (A–B) or #301703 (C).

For analysis of IL-1α expression in the pigs, qRT-PCR was first carried out on total RNA from cultured keratinocytes. In five of the six hITGB1-transgenic pigs a significantly enhanced IL-1α mRNA level was detected ranging from a 2- to 6-fold increase relative to the control pig #2990 (**Fig. 6A**). Further enhancement of IL-1α expression was detected in hITGA2-transgenic animals. For the three animals that were analyzed, the IL-1α mRNA levels were increased from 6- to 27-fold relative to three distinct control pigs (**Fig. 6B**). Notably, this level of induction was directly correlated with the α2 integrin expression level (compare with **Fig. 5E**), indicating that the induced cytokine response was triggered by the transgenic expression of α2 integrin. Assessment of cellular IL-1α release was achieved by an ELISA-based approach using an antibody specific for porcine IL-1α. Medium conditioned for 14 hours showed a 2- to 5-fold increment in secreted IL-1α by cells expressing β1 integrin compared to the average value obtained with medium conditioned by keratinocytes from the non-transgenic controls, pigs #301703 and #301706 (**Fig.–6C**). Together, these findings documented an increased expression of IL-1α in hITGB1- and hITGA2-transgenic porcine keratinocytes.

**Figure 7:**
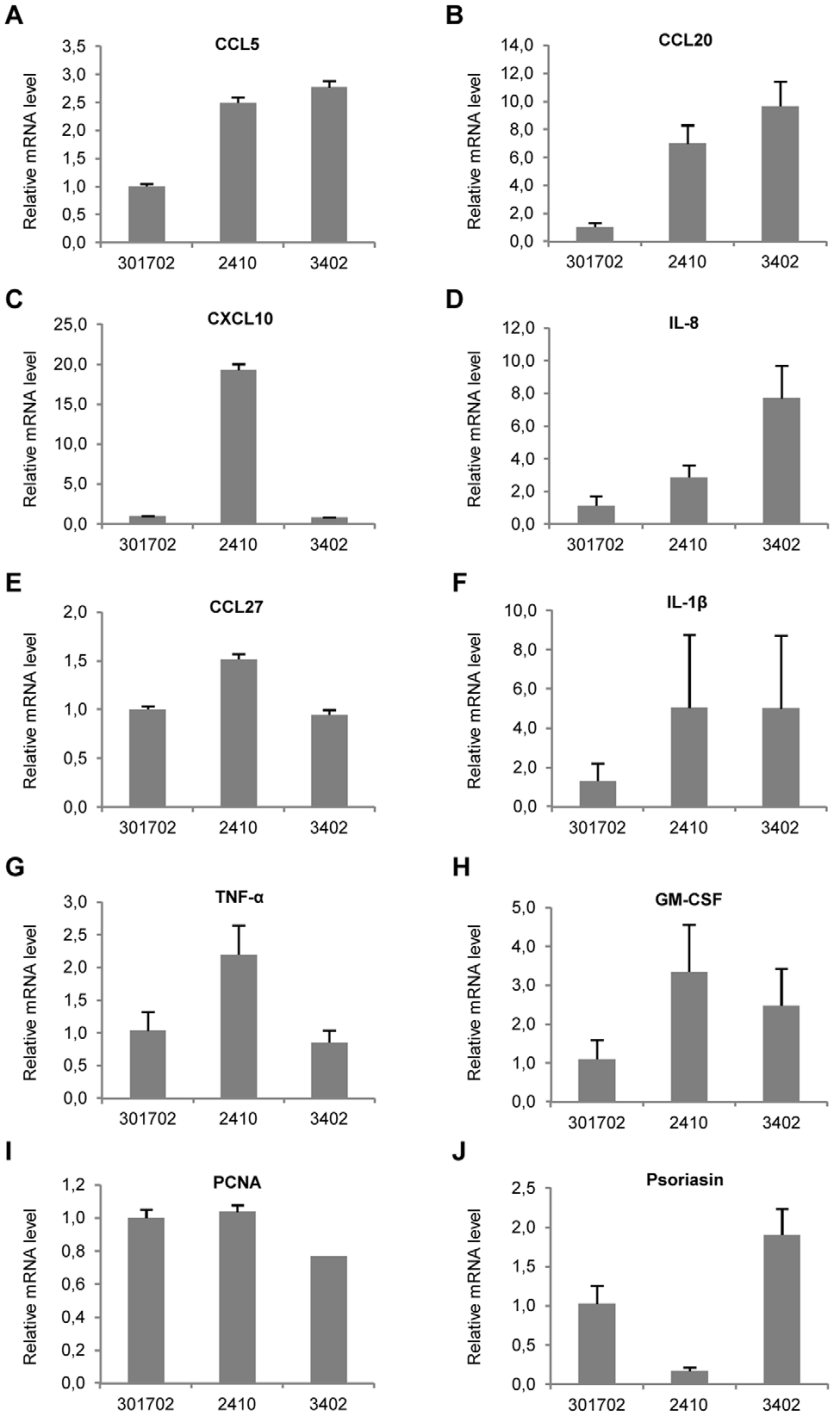
Altered cytokine and chemokine profile in hITGB1-transgenic animals. Quantitative RT-PCR directed against ten molecular markers of skin-inflammation performed on total RNA extracted from punch biopsies taken from the non-transgenic pig #301702 and the hITGB1-transgenic pigs #2410 and #3402 at age 14 months, normalized to endogenous GAPDH. Data are technical triplicates and presented as mean values ± standard deviations. CCL5, CCL20, and CCL27 (Chemokine (C-C motif) ligand 5, 20, and 27, respectively); CXCL10 (chemokine (C-X-C motif) ligand 10); IL-8 and IL-1β (interleukins 8 and 1β); TNF-α (tumor necrosis factor alpha); GM-CSF (granulocyte-macrophage colony-stimulating factor); PCNA (proliferating cell nuclear antigen).

To assess if the general inflammatory profile was altered in the transgenic animals, a panel of ten crucial markers of inflammation was analyzed by qRT-PCR on total RNA derived from skin biopsies taken from the back of the hITGB1-transgenic pigs #2410 and #3402 and compared to a skin sample from the wildtype control #301702 (**Fig. 7**). The chemokines chemokine (C-C motif) ligand 5 (CCL5), CCL20, chemokine (C-X-C motif) ligand 10 (CXCL10), and IL-8 are released by several cell types in the early phase of inflammation and recruit lymphocytes and granulocytes to the site of inflammation. Compared to the wildtype control pig, the level of mRNA encoding these factors was elevated from 2- to 19-fold in the two transgenic animals (**Fig. 7A–D**), whereas the level of the skin-specific chemokine CCL27 was only moderately upregulated in one of the pigs (**Fig. 7E**). The level of expression of the cytokines IL-1β, tumor necrosis factor alpha (TNF-α) and granulocyte-macrophage colony-stimulating factor (GM-CSF) was increased 2- to 5-fold, as judged by qRT-PCR (**Fig. 7F–H**), whereas no changes were seen in the case of proliferating cell nuclear antigen (PCNA) (**Fig. 7I**). The expression of the antimicrobial and chemotatic protein psoriasin was markedly decreased in pig #2410 but moderately increased in pig #3402 (**Fig. 7J**). In summary, these results indicate that a broad perturbation of cutaneous cytokine and chemokine expression was inflicted by subrabasal expression of β1 integrin.

**Figure 8:**
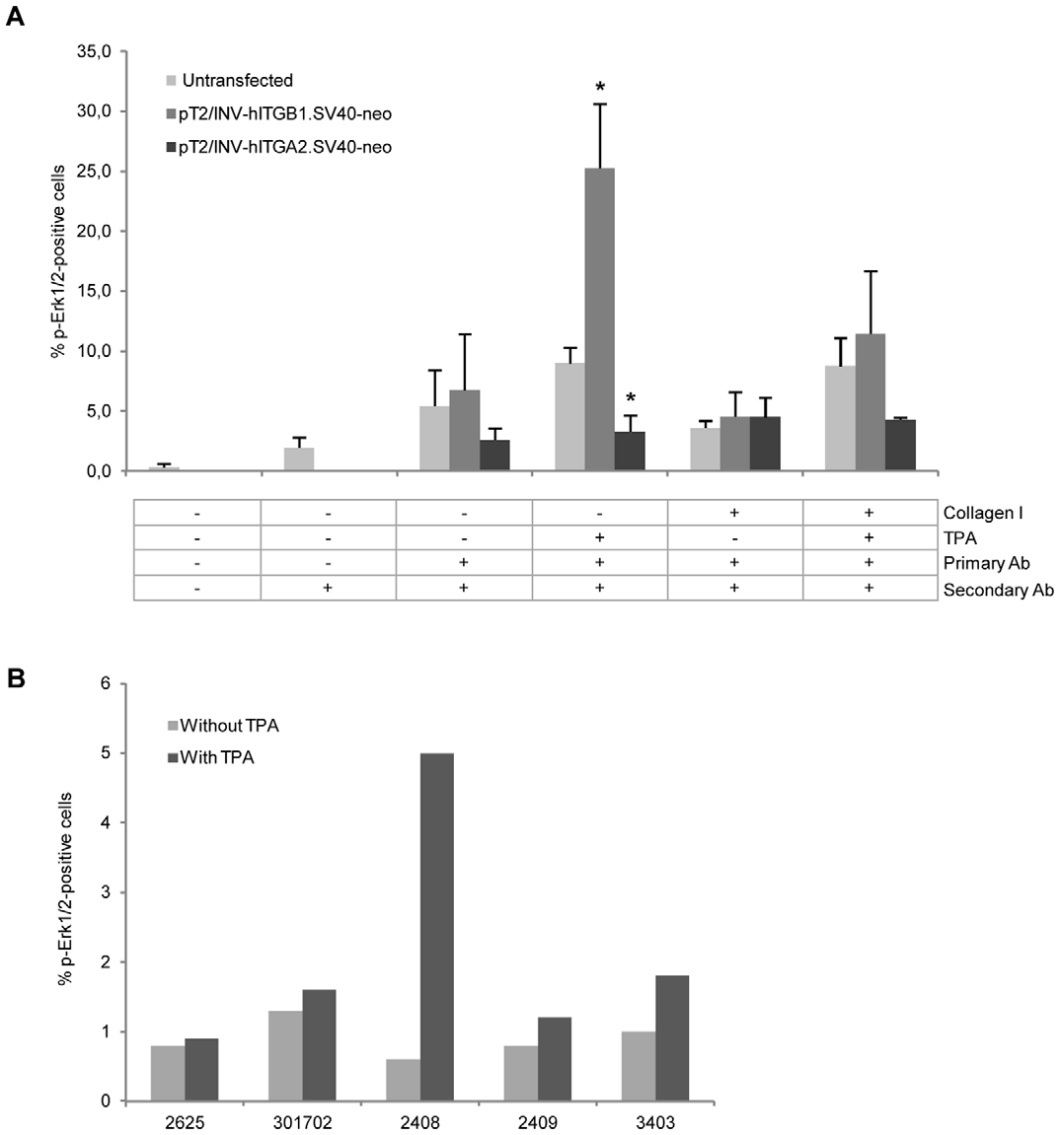
Erk1/2 phosphorylation in TPA-stimulated hITGB1-transgenic keratinocytes but not in hITGA2-transgenic keratinocytes. (**A**) Naïve and hITGB1- or hITGA2–expressing HaCaT cells were grown to subconfluency on uncoated or collagen I-coated plates. After two days of incubation in serum-depleted medium, one subset was stimulated with 100 ng/mL TPA for 10 min, after which all subsets were stained with a phosphor-Erk1/2 specific mouse mAb (E10). Increased levels of pERk1/2, indicative of increased Erk/MAPK activation, could be detected in stimulated β1 integrin-expressing HaCaT cells, whereas an inhibition upon TPA stimulation was apparent in α2 integrin-expressing cells. Asterisks (*) indicate statistical significance relative to naïve HaCaT cells. (**B**) TPA-induced activation of Erk1/2 phosphorylation in transgenic keratinocytes. Cultured hITGB1-transgenic keratinocytes were treated as described under (**A**). In case of pig #2408, a marked difference in phosphorylated Erk1/2 levels was detected by comparing TPA-stimulated and un-stimulated cells. Data are presented as mean values ± standard deviations.

Phosphorylation of Erk1/2 (pErk1/2) is a prerequisite for the nuclear import and activity of Erk1/2 during induction of the Erk/MAPK pathway. Elevated levels of nuclear pErk1/2 are evident in psoriatic lesions and in mouse keratinocytes engineered to express the hITGB1 gene [49]. To study the phosphorylation of Erk1/2, we first investigated the effect of stably expressing the hITGB1 and hITGA2 genes in HaCaT keratinocytes. Naïve and transgenic cells were cultured in serum-depleted medium in dishes that were either coated with collagen I or left uncoated. One subset of cells was subsequently treated with the protein kinase C (PKC) activator 12-O-tetradecanoyl phorbol-13-acetate (TPA), after which all cells were trypsinized, immunostained with a pErk1/2-specific antibody, and analyzed by flow cytometry. Exposure to TPA increased the number of naïve HaCaT cells that were positive for pErk1/2 less than 2-fold, whereas the number of pErk1/2-positive cells carrying the transposon-embedded hITGB1 gene was increased 4-fold by TPA induction, leading to pErk1/2 labeling of more than 25% of the cells (**Fig. 8A**). In contrast to cells stably transfected with the hITGB1 gene, TPA did not lead to increased phosphorylation of Erk1/2 in hITGA2-transgenic HaCaT cells. In fact, the pathway seemed to be down-regulated compared to naïve cells, resulting in approximately half the proportion of cells that were positive for pErk1/2 relative to non-transfected cells (**Fig. 8A**). Together, these findings suggested that keratinocytes with engineered expression of β1 integrin were increasingly sensitive to external stimuli and that the response to extracellular stimuli was differentially affected by engineered expression of β1 and α2 integrin. We therefore analyzed the level of phosphorylated Erk1/2 in keratinocytes derived from β1 integrin-transgenic pigs. We succeeded to prepare primary keratinocytes from three pigs for this analysis. There was no remarkable change in the level of pErk1/2 in untreated or TPA treated cells from each control pig, whereas for pig #2408 an 8-fold increase in the number of positive cells was evident after TPA stimulation.

**Figure 9:**
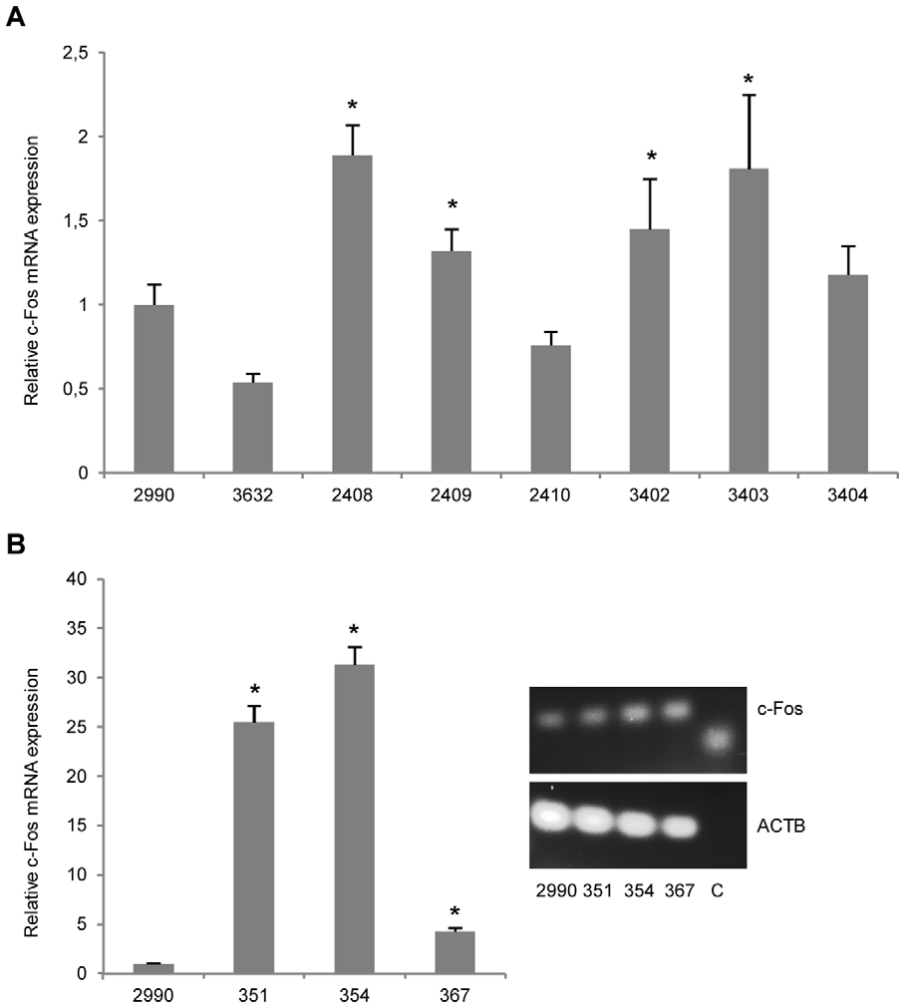
Elevated c-Fos mRNA expression levels in un-stimulated transgenic pig keratinocytes. (**A**) Quantitative RT-PCR on total mRNA extracted from cultured keratinocytes derived from non-transgenic pigs (#2990 and #3632) and the six hITGB1-transgenic pigs, normalized to endogenous β-actin. A modest increment of endogenous c-Fos mRNA was detectable in four out of the six transgenic keratinocytes compared to the levels in control pigs #2990 and #3632. (**B**) Quantification of c-Fos mRNA levels in hITGA2-expressing keratinocytes was performed as described in (A). Images of gel-electrophoresed qRT-PCR products are depicted (insert); C indicates the water control. A substantial increase of c-Fos mRNA levels was recorded in the transgenic keratinocytes derived from the three hITGA2-transgenic pigs compared to the levels in control pig #2990. Data are presented as mean values ± standard deviations. Asterisks (*) indicate statistical significance compared to #2990.

Finally, we inspected the expression of the immediate early gene c-Fos in transgenic keratinocytes. C-Fos is a downstream effector of p38/MAPK and Erk/MAPK activation and dimerizes with c-Jun to form the transcription complex AP-1. AP-1 up-regulates a variety of genes involved in important biological processes including cell proliferation, differentiation, survival, and repair mechanisms as well as embryonic development [51], [52]. In the stratum basale and stratum spinosum, c-Fos is normally not (or only slightly) expressed [53]. We speculated that transgenic integrin expression could have an impact on the expression of c-Fos with possible implications for skin homeostasis. Levels of keratinocyte c-Fos mRNA was increased in all hITGB1-transgenic pigs, except one (pig #2410). However, the enhancement was small (at most 2.5-fold above the average level measured for the two control pigs) and varied considerably among the animals (**Fig. 9A**). In contrast, in case of the keratinocytes derived from the three α2 integrin-transgenic pigs that were analyzed, we observed a considerable induction of c-Fos, resulting in at most a 31-fold enhancement in the level of c-Fos mRNA (**Fig. 9B**). These findings suggest that expression of transgenic α2 integrin, and to a minor degree β1 integrin, triggers cellular signaling pathways that involve the induction of c-Fos.

In summary, we conclude that molecular markers of induced skin inflammation could be identified in keratinocytes from pigs that were transgenic for either human β1 or α2 integrin expressed from the involucrin promoter exclusively in the suprabasal skin layer. Although both integrins cause enhanced expression and secretion of IL-1α, we also note that β1 integrin, and not α2 integrin, increases the phosphorylation of Erk1/2. Furthermore, α2 integrin, and only to a smaller degree β1 integrin, triggers induced expression of c-Fos. These findings suggest that different pathways of cellular signaling (potentially the Erk/MAPK and p38/MAPK pathways) are differentially affected by the transgenes with possible implications for the longer-term phenotype of the animals.

## Discussion

With establishment of pig cloning by somatic cell nuclear transfer it is now possible to produce transgenic animals from genetically engineered somatic cells. Recently, we established the use of SB DNA transposon-based vectors for development of transgenic pigs by DNA transposition in cultured cells serving as nuclear donors in handmade cloning [54]. In the present report, we explore this approach for generating pig models of skin inflammation. We established Göttingen minipigs carrying human transgenes encoding either β1 or α2 integrin and demonstrated subrabasal expression of the exogenous integrins.

To ensure that transgene transcription was confined to the subrabasal layers, the integrin expression cassette was driven by the human involucrin promoter. We successfully generated six unique hITGB1 pigs containing between one and six transposon insertions. In case of the hITGA2 pigs, however, six of seven pigs were found to be genetically identical, suggesting that they were derived from a single clone out of approximately 30 pooled G418-resistant donor cells. We have previously observed this phenomenon [54] which may reflect a large selection pressure in effect during *in vitro* growth of somatic cells or during blastocyst and embryo development. It is currently not known whether any toxicity related to expression of the transgene or the SB transposase, or to the transposition process itself, may cause certain clones to exhibit growth advantages and increased survival relative to other clones.

The aggregation and ligation of membrane-bound integrins play a crucial role for strong and persistent activation of members of the large group of receptor tyrosine kinases (RTKs). The group of RTKs, including the EGFR family, serves as receptors for extracellular ligands including cytokines and growth factors and, upon activation, activates cellular signaling potentially through the MAPK pathway. Although mitogen-activated RTKs mediate strong– and integrin ligation only weak– MAPK activation, they are both temporary. In fact, induction of the MAPK pathway is diminished within one hour of integrin ligation [9], [49]. However, when such pathways of activation act in synergy, the activation of MAPK signaling is sufficient to induce cell growth [55]. Integrins and RTKs are known to co-localize in higher-order complexes which may support prolonged MAPK activation by potentiating phosphorylation, protecting from dephosphorylation, or preventing internalization of the RTKs [56], [57], [58]. Interestingly, β1 integrin may induce EGFR phosphorylation and thereby activate the MAPK pathway even in the absence of EGFR ligands [9], [11]. Several integrins, including β1 integrin, have been shown to cluster with other membrane receptors during cell-to-cell adhesion 59,60,61. Thus, it is conceivable that integrin ligation caused by cell-to-cell interactions, in conjunction with integrin-directed activation of RTKs, elicit a strong activation of the MAPK pathway.

Subrabasal overexpression of β1 integrin in mice has been shown to entail keratinocyte hyperproliferation and disturbed differentiation. As a direct response to this, variable degrees of perturbations of the epidermis were evident causing chronic inflammation and in some instances conditions reminiscent of adult plaque psoriasis [14], [62]. Such visible cutaneous abnormalities either arose spontaneously or where induced by physical irritation (e.g. tape stripping) or topically administered chemicals (e.g. phorbol esters like TPA). The mechanism by which subrabasal integrins induce hyperproliferation and altered differentiation is still obscure. However, subrabasal MAPK activation coincides with β1 integrin expression. Moreover, β1 integrin ligation on the surface of cultured keratinocytes leads to MAPK induction, suggesting that the capability of β1 integrins to induce signal transduction plays a role during the induction of skin inflammation in the mouse [49].

Confocal microscopy of keratinocytes derived from hITGB1 transgenic pigs demonstrated membrane localization of the transgenic protein and the accumulation of β1 integrin at cell-to-cell interaction points. To investigate the potential activation of the MAPK pathway in transgenic animals, we first studied the level of Erk1/2 phosphorylation in TPA-stimulated HaCaT keratinocytes stably expressing β1 or α2 integrin. Notably, only β1 integrin triggered an elevated level of pErk1/2 in HaCaT cells, and the effect of β1 integrin was therefore explored in keratinocytes from β1 integrin-transgenic pigs. At least for one of three analyzed pigs (#2408) we could monitor a marked increase in the percentage of cells positive for phosphorylated Erk1/2 (also referred to as mitogen-activated protein kinase 3 and 1), suggesting that the MAPK pathway was activated upon stimulation with TPA. Similarly, the Erk/MAPK pathway was activated upon TPA stimulation of keratinocytes derived from α2β1 integrin double-transgenic mice [63], suggesting that constitutive expression of integrins in subrabasal keratinocytes rendered the keratinocytes hypersensitive to external stimuli. The fact that the level of pErk1/2 was unaffected by expression of α2 integrin, as based on our findings in TPA-stimulated HaCaT cells, suggests that β1, but not α2, integrin plays a crucial role for the activation of the Erk/MAPK pathway in this context. Previous work has demonstrated that the cytoplasmic tail of α2 integrin mediates signals via p38/MAPK [64], [65]. This effect is probably stimulated through the small G-proteins Cdc42 and Rac1 [66], a route of signaling which is not stimulated by TPA. Interestingly, several reports have shown that p38 has an inhibitory effect on Erk1/2 which can be mediated either through p38-directed stimulation of protein phosphatase 2 (PP2A) [67], [68] or via a direct interaction between p38 isoforms and Erk1/2 [69], [70].

To further define the inflammatory phenotype of keratinocytes from transgenic pigs, we analyzed transgenic keratinocytes for potential induction of IL-1α as a marker for an altered inflammatory profile. The expression of IL-1α was found to be elevated in all transgenic pigs, indicating that both β1 and α2 integrin gave rise to transcriptional induction of IL-1α. However, the enhancement was far more pronounced for α2 integrin pigs relative to β1-transgenic animals. This suggests that overexpression of α2 integrin induces cellular signaling conveyed by routes different from β1 integrin overexpression. IL-1α is a very potent proinflammatory cytokine which is constitutively produced by epithelial cells, especially keratinocytes, leading to activation of the NF-κB pathway and recruitment of activated mononuclear cells– among a range of effects. Following skin wounding IL-1α is discharged from intracellular storage vesicles [71]. Secreted IL-1α contributes to keratinocyte hyperproliferation by EGFR-dependent activation of the Erk/MAPK pathway and has been shown to induce inflammatory conditions in mouse and human epidermis [72], [73]. Under normal conditions the activity of IL-1α is tightly regulated [74] by mechanisms that do not depend on ECM-induced integrin ligation [49]. However, there are numerous examples of signal co-operativity between integrins and IL receptors, suggesting that integrins may assist in intensifying cytokine signaling [75], [76]. Indeed, integrin-mediated transcriptional activation of IL-1α has previously shown to involve Erk1/2 or NF-κB activation [50] although the exact mechanism by which the release of IL-1α is stimulated is still uncertain. The demonstration of increased IL-1α production in β1 and α2 integrin-transgenic pigs supports the notion that increased integrin signaling correlates with enhanced levels of porcine cytokine production and an altered inflammation profile.

To clarify if the ectopic β1-integrin expression induced a general alteration of the cytokine and chemokine profile in the transgenic animals, we analyzed the mRNA levels of a series of inflammation markers in biopsies of untreated skin from two hITGB1-transgenic animals. The inflammatory response instigated by subrabasal integrin expression has previously shown to comprise T_H_1- and T_H_17-related cytokines like IL-1β, IFN-γ, GM-CSF and TNF-α [62]. Importantly, the T_H_1 cytokine signature has been associated with the pathogenesis of psoriasis [77]. Notably, for all the cytokines investigated, except TNF-α, the level of mRNA was upregulated in both transgenic animals compared to an age-matched control. The level of TNF-α mRNA, in contrast, was only enhanced in one of the two pigs. A general upregulation of relevant chemokines was also observed. In addition, an increased level of psoriasin was detected in one of the two transgenic pigs. Psoriasin has been identified as a marker for hyperproliferative and inflammatory skin disorders such as psoriasis and atopic dermatitis [78], [79]. Overexpression of CXCL10 has also been linked to psoriasis [80]. It is predominately activated by IFN-γ, and recruits activated T-lymphocytes and NK cells. Notably, there was a significant difference in the expression level of psoriasin and CXCL10 in the two transgenic animals. Interestingly, it has previously been shown that psoriasin expression is transcriptionally suppressed by IFN-γ [81]. Taken together, the expression profile of inflammatory markers suggests that a wide-spread dysregulation of the immune system was prompted by the ectopic expression of β1-integrin. Peculiarly, the expression pattern seemed to vary between the two hITGB1-transgenic pigs, suggesting that the exact expression profile of the integrin itself played a role on downstream effects.

The expression of c-Fos is induced through both the p38/MAPK and the Erk/MAPK signaling pathways [82], and this marker is therefore, like IL-1α, a more general indicator of altered signaling caused by phosphorylation of transcription factors that bind c-Fos enhancer elements [83]. We therefore measured the levels of c-Fos mRNA in transgenic keratinocytes. Whereas the increase was only moderate in hITGB1-transgenic keratinocytes (a significant increase in four of six analyzed animals), we observed a solid enhancement of c-Fos mRNA in all three hITGA2-transgenic animals that were analyzed. Although the total number of animals was small, these data support the notion that different signaling patterns were activated in the two groups of transgenic animals.

To date we have not registered any visible epidermal abnormality, or psoriasis-like phenotype, in any of the hITGB1- or hITGA2-transgenic pigs, neither by direct examination of the skin nor by analysis of skin morphology by staining of skin section. However, it is widely accepted that environmental influences, in conjunction with genetic components, play an important role in the pathogenesis of inflammatory diseases such as psoriasis. In α2/β1 integrin double-transgenic mice, skin irritation led to a chronic condition resembling psoriasis with persistent hyperplasia, cutaneous influx of CD4^+^ and CD8^+^ T-lymphocytes and secretion of pro-inflammatory cytokines like TNF-α, IFN-γ, IL-1β and IL-6 [62]. Based on our molecular analysis of central indicators of inflammation findings, we believe that the transgenic pigs may be pre-disposed for development of an exacerbated and potentially chronic inflammation by provocation. Ongoing studies have been designed to address the potential development of chronic plaque lesions in the transgenic pigs as a response to mechanically or chemically induced stress.

In conclusion, we have created Göttingen minipigs transgenic for the human integrins β1 and α2 and have demonstrated efficacious transgene expression in skin as well as induced inflammatory signaling in transgenic keratinocytes. We believe that such porcine models of skin inflammation will contribute to a better understanding of the pathogenesis of cutaneous diseases and will be of great potential in studies aiming at the development and refinement of topical therapies for cutaneous inflammation including psoriasis.

## Materials and Methods

### Ethics Statement

All animal procedures were approved by the Danish Animal Experiments Inspectorate (license no. 2006/561-1230).

### Plasmids and vector construction

SB transposon-based vector constructs were generated based on the plasmid vector pT2/SV40-neo which has been previously described [47]. An expression cassette containing the eGFP ORF driven by the cytomegalovirus (CMV) promoter was PCR-amplified in two steps from pEGFP.N1 (Clontech), allowing the insertion of a unique linker between the eGFP gene and the promoter. Subsequently, the CMV-eGFP fragment was combined with the bGH polyA signal derived from pTOPO (Invitrogen, Paisley, UK) in an overlap extension PCR creating a fragment with an EcoRI-XbaI linker in each terminus. The PCR product was digested and inserted into pT2/SV40-neo digested with either EcoRI or XbaI (which cuts upstream and downstream, respectively, of the SV40-neo cassette). This gave rise to four constructs (pT2/CMV-eGFP-bGHpA.SV40-neo, pT2/bGHpA-eGFP-CMV.SV40-neo, pT2/SV40-neo.CMV-eGFP-bGHpA and pT2/SV40-neo.bGHpA-eGFP-CMV) in which individual components could be easily exchanged. The eGFP ORF of pT2/CMV-eGFP-bGHpA.SV40-neo was released with NotI and substituted with either the hITGB1 cDNA sequence (encoding β1 integrin) PCR-amplified from pCMV6-XL5 (SC111935, Origene, Rockville, MD, USA) or the hITGA2 cDNA sequence (encoding α2 integrin) amplified from pCMV6-XL4 (SC118747, Origene, Rockville, MD, USA), generating the constructs designated pT2/CMV-hITGB1-bGHpA.SV40-neo and pT2/CMV-hITGA2-bGHpA.SV40-neo, respectively. The CMV promoter was subsequently released by AscI digestion and replaced with the PCR-amplified involucrin (INV) promoter from pH3700-pL2 (kindly provided by Rikke Christensen, Dept. of Biomedicine, University of Aarhus, Denmark), leading to pT2/INV-hITGB1-bGHpA.SV40-neo and pT2/INV-hITGA2-bGHpA.SV40-neo. pCMV-SB100X has been previously described [84].

### Cells

The murine fibroblast cell line NIH3T3 and human keratinocyte cell line HaCaT were cultured in Dulbeccós modified Eaglés medium, DMEM (Lonza, Verviers, Belgium) with 10% fetal calf serum, FCS (Lonza, Verviers, Belgium). Established Göttingen primary fibroblasts were maintained in DMEM with 15% FCS. All media were supplemented with 100 U/mL penicillin, 0.1 mg/mL streptomycin and 265 mg/l L-glutamine and all cell were maintained at 37°C, 5% CO_2_. Outgrowth and expansion of primary fibroblasts and keratinocytes was achieved from explants derived from pig ear-biopsies. The epidermis was sectionally isolated and the explants were placed in 25-cm^2^ culture flasks (TPP, Trasadingen, Switzerland) and incubated bottom-up O/N at 37°C, 5% CO_2_. Fibroblast outgrowth was promoted in AmnioMAX-C100 (Gibco, Invitrogen, Paisley, UK). Outgrowth and expansion of keratinocytes was achieved in 15% FCS DMEM containing the additives as described above plus 10 ng/mL EGF (Gibco, Invitrogen, Paisley, UK), 50 µM gentamycin and 0,4 µg/mL hydrocortisone (Sigma-Aldrich, St. Louis, MO, USA) at 37°C, 5% CO_2_ for 7–10 days, allowing keratinocytes to migrate from the explants. Hereafter, the medium was substituted with epidermal growth factor (EGF) and bovine pituitary extract (BPE) containing serum-free keratinocyte medium, K-SFM (Gibco, Invitrogen, Paisley, UK).

### Handmade cloning (HMC) and transfers

HMC was performed as described before [29], [31]. Briefly, cumulus cells were removed from matured cumulus-oocyte complexes (COCs) by treatment with hyaluronidase. After partial digestion of zona pellucida, oriented bisection of oocytes was performed to remove nuclei. Each cytoplast without polar body attached with a single trangenic fibroblast was fused in fusion medium (0.3 M mannitol, 0.1 mM MgSO_4_ and 0.01% [w/v] PVA) in a fusion chamber (BTX microslide 0.5 mm fusion chamber, model 450; BTX, San Diego, CA, USA) with a single direct current (DC) impulse of 2.0 kV/cm for 9 μs. One hour later, each cytoplast-somatic cell pair was fused with another cytoplast in activation medium (fusion medium with 0.1 mM CaCl_2_) by a single DC pulse of 0.86 kV/cm for 80 μs. After incubation in porcine zygote medium 3 (PZM-3) supplemented with 5 µg/ml cytochalasin B, 10 µg/ml cyclohexinmide for 4 h, the reconstructed embryos were cultured in PZM-3 medium for another 6 days to develop into transgenic blastocysts. Blastocysts at day 5 and 6 were surgically transferred into recipient sows [31]. All animal procedures were approved by the Danish Animal Experiments Inspectorate (license no. 2006/561–1230).

### Immunostaining

For immunocytochemistry, HaCaT cells stably transfected with pT2/INV-hITGB1.SV40-neo or pT2/INV-hITGA2.SV40-neo were seeded in slideflasks (Nunc A/S, Roskilde, Denmark) with a density of 2×10^5^ cells/flask. After 24–48 h the cells were fixed in 4% formalin for 5–10 min, washed in PBS and blocked in 0.5% BSA, 0.3% Triton-X100 PBS for 30 min at ambient temperatures. Subsequently, the cells were incubated O/N at 4°C with the mouse anti-human mAb Alexa488-P5D2 (kindly provided by Uffe Birk Jensen, Dept. of Biomedicine, University of Aarhus, Denmark) in 0,5% BSA, 0,3% Triton-X100 PBS for hITGB1-expressing cells and with the mouse anti-human mAb P1E6 (Santa Cruz Biotechnology Inc, CA, USA) for hITGA2-expressing cells. The cells were washed in 0.05% Tween20 TBS. and the mAb P1E6 treated cells were additionally incubated with an Alexa-488 goat anti-mouse IgG secondary antibody (Invitrogen, Paisley, UK ) for 1h at room temperature and once again washed in 0.05% Tween20 TBS. The slides were mounted in mounting medium (Vectashield, Vector Laboratories Inc, Burlingame, CA) containing 1.5 µg/mL DAPI and visualized with a Leitz DMRB microscope (Leica Microsystems CMS GmbH, Wetzlar, Germany). Skin explants from α2 or β2 integrin-transgenic pigs were placed in slideflasks from which primary keratinocytes were expanded. After 2 weeks the explants were removed and the slides were stained as described above. For immunohistochemistry, ear biopsies from β2 integrin-transgenic pigs were embedded in OCT (Sakura Finetek Europe, The Netherlands) and snap frozen in liquid N_2_ and sections of 6 µm hereof were cut on a cryostat (Microm HM 500 M, Microm International GmBH, Waldorf, Germany). Slides were treated as described above.

### Flow Cytometry

For flow cytometry assessment of hITGA2 and hITGB1 expression, transgenic and non-transgenic primary porcine keratinocytes were trypsinized and fixed in 4% formalin for 10 min. The populations were split into two tubes. One part was blocked in PBS with 0.5% BSA at 4°C, 30 min, the second part was additionally permeabilized with 0.2% saponin (Sigma-Aldrich, St. Louis, MO, USA). All samples were washed in TBS +0.5% Tween and stained with the Alexa 647-conjugated anti-K14 mAb LL002 (kindly provided by Uffe Birk Jensen, Dept. of Biomedicine, University of Aarhus, Denmark) at 1∶100 for 4°C, 60 min. Additionally, staining for α2 integrin was accomplished with the monoclonal anti-α2 primary antibody, P1E6 (Santa Cruz Biotechnology, CA, USA) at 1∶50 for 4°C, 60 min., washed and subsequently incubated with the highly cross-adsorbed Alexa 488 goat anti-mouse IgG, A11029 (Invitrogen, Paisley, UK) at 1∶400 for 4°C, 60 min. Staining for β1 integrin was achieved with the Alexa 488-conjugated monoclonal anti-β1 antibody, P5D2 1∶100 for 4°C, 60 min. The cells were washed and resuspended in 300 µL PBS (no MgCl_2_, no CaCl_2_). Subsequently, 10,000 events were analyzed on a BD FACSAria III machine using BD FACSDiva and FlowJo software. For the evaluation of phosphorylated Erk1/2 in HaCaT cells, 5×10^5^ cells were seeded on uncoated or collagen I-coated P10 plates in 10% FCS DMEM. After 24h incubation the medium was exchanged with serum-free DMEM and the cells were incubated for additional 48 hours. Subsequently, appropriate plates were incubated with 100 ng/mL TPA (Sigma-Aldrich, St. Louis, MO, USA) for 8 min. All plates were trypsinized, and the cells were fixed in 90% ice-cold methanol for 30 min on ice. All samples were washed in TBS +0,05% Tween and blocked in PBS with 0.5% BSA on ice, 30 min; hereafter, p-Erk1/2 was bound with the mouse mAb E10 (Cell Signaling Technology, MA, USA) at 1∶800 for 1 h on ice. The samples were washed and incubated with the Alexa 488 goat anti-mouse IgG, A11029 at 1∶400 on ice for 1 h. Transgenic and non-transgenic porcine keratinocytes expanding from skin explants were induced with 100 ng/mL TPA for 8 min., trypsinized and processed as described for HaCaT cells.

### Confocal microscopy

hITGB1-transgenic or control pig keratinocytes were seeded at a density of 10^4^/chamber in a 8-well poly-L-Lysine coated 1µ-slide plate (Ibidi, Munich, Germany). After 24 h incubation, the cells were fixed, permeabilized and stained with the Alexa488-P5D2 mAb as described above. The cells were visualized utilizing a 488 nm line of a multiline argon laser (detection of Alexa-488) and the 405 nm line of a 405–30 nm diode laser (detection of DAPI) in a confocal laser scanning microscope (LSM 710, Zeiss, Jene, Germany) using 63″ oil-immersion objective with a numerical aperture of 1.4.

### IL-1α ELISA

hITGA2- and hITGB1-transgenic keratinocytes as well as control pig keratinocytes were grown in 3 mL complete K-SFM in uncoated flasks for 14 h, after which the conditioned medium was harvested. Secreted porcine IL-1α levels were measured by ELISA (Cusabio Biotech CO Ltd., Wuhan, Hubei, China) and correlated to the number of viable cells determined with a NucleoCounter (ChemoMetec A/S, Allerød, Denmark) and in a Bürker-Türk cell counting chamber.

Additional details on materials and methods are provided in the ‘Supplementary Materials and Methods S1’ file that is available online from the PLoS One web site.

## Supporting Information

Figure S1 **Karyotyping of the six hITGB1 transgenic pigs revealed no gross abnormalities.** Karyotyping of mitotic arrested fibroblasts from the six hITGB1 transgenic pigs showed a normal diploidic karyotype with 36 autosomal and 2 sex chromosomes for all pigs. Based on the karyogram no gross chromosomal abnormalities could be detected. A representative image of the DAPI stained karyogram for pig #3404 is shown.
(TIF)

Figure S2 **Histological** **examination of skin sections from hITGB1 transgenic pigs by** **haematoxylin and eosin** **staining.** Skin biopsies from the six hITGB1-transgenic and control pigs were taken at the age of 1, 5 and 14 months. The biopsies were embedded in OCT, snap frozen in liquid nitrogen, sectioned into 6 µm slices and H&E-stained. No change in skin morphology could be detected in any of the six hITGB1-transgenic pigs over the period of 14 months. Representative pictures are shown for control #2990 (a–b), control #301706 (c) and hITGB1-transgenic pig #3404 (c–e).
(TIF)

Figure S3 **Quantification of hITGA2 mRNA by qRT-PCR in tissues from hITGA2-transgenic pig #554.** Tissue biopsies from the sacrificed pig #554 and an age-related wildtype pig were grinded after which total RNA was extracted and employed for hITGA2-directed qRT-PCR, normalized to endogenous β-actin mRNA levels. No hITGA2 was detected in any of the samples from the non-transgenic control pig. Obtained values in pig #554 are shown relative to the level detected in one of the skin samples (skin A) which is set to 1. A statistically significant (p<0.0001) expression was seen in all pig #554 skin samples relative to internal tissues. Skin samples A–F were taken from different locations on pig #554. Data are presented as mean values ± standard deviations.
(TIF)

Materials and Methods S1 Transposition assays and generation of donor cells for HMC, H&E staining, PCR and qRT-PCR, Identification of SB insertion sites by LDI-PCR and sequencing, Southern blot analysis, Karyotype analysis, and Western blot analysis.
(DOCX)

Table S1 **Summary of cloning efficiencies obtained with hITGB1-transgenic fibroblasts.**
(TIF)

Table S2 **Summary of genotyping results obtained in hITGB1-transgenic pigs.**
(TIF)

Table S3 **Summary of cloning efficiencies obtained with hITGA2-transgenic fibroblasts.** The only surviving pig is indicated with an asterisk (*).
(TIF)

Table S4 **Summary of genotyping results obtained in hITGA2-transgenic pigs.**
(TIF)

Table S5 **Summary of re-cloning efficiencies obtained with original hITGA2-transgenic fibroblasts.**
(TIF)
