# Supplementary figures and images for: Development of Transgenic Cloned Pig Models of Skin Inflammation by DNA Transposon-Directed Ectopic Expression of Human β1 and α2 Integrin
Source: PLoS One. 2012 May 10;7(5):e36658. doi: 10.1371/journal.pone.0036658 (PMC3349713; doi:10.1371/journal.pone.0036658)

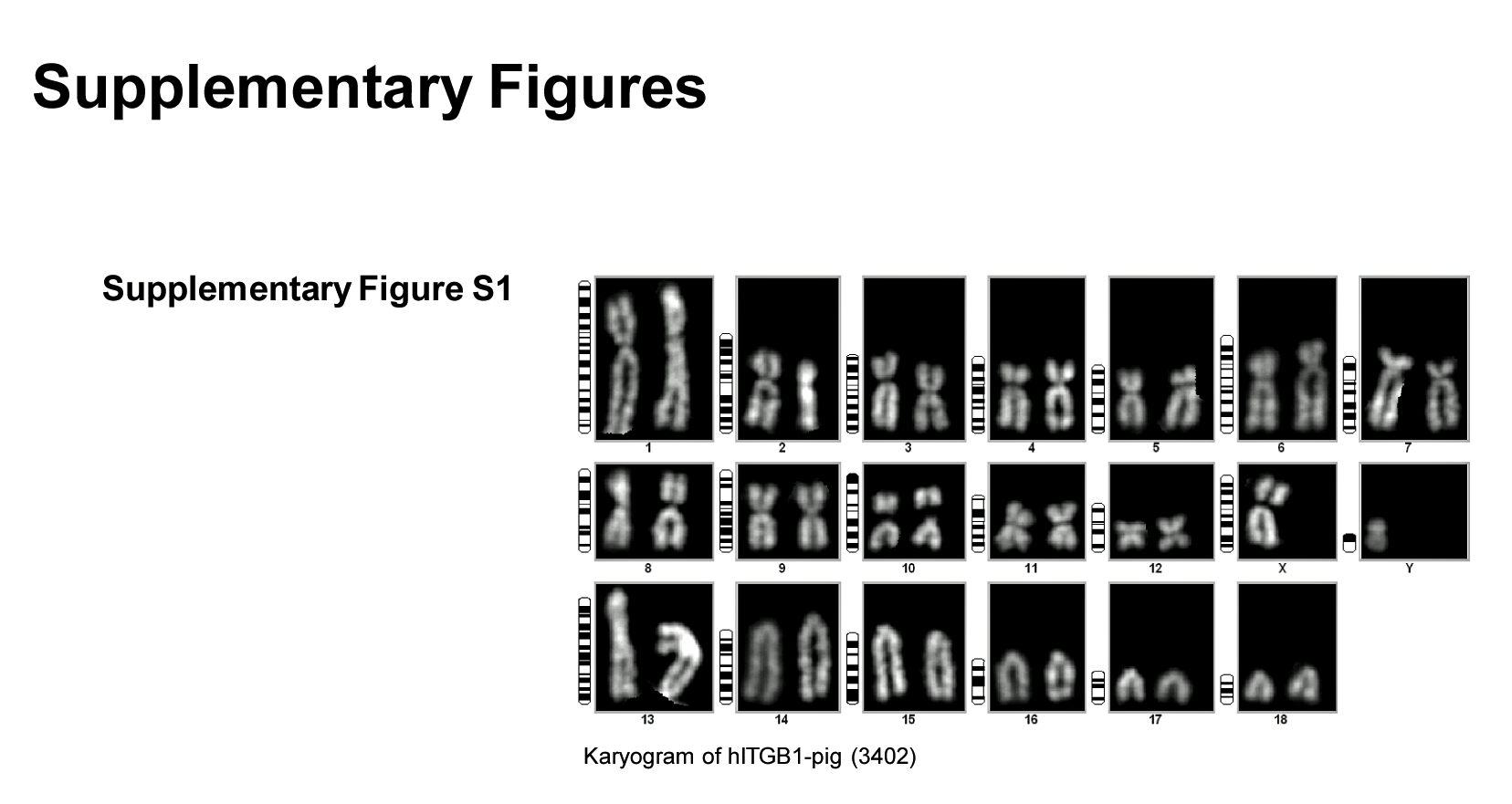

Supplement: Figure S1 — Karyotyping of the six hITGB1 transgenic pigs revealed no gross abnormalities. Karyotyping of mitotic arrested fibroblasts from the six hITGB1 transgenic pigs showed a normal diploidic karyotype with 36 autosomal and 2 sex chromosomes for all pigs. Based on the karyogram no gross chromosomal abnormalities could be detected. A representative image of the DAPI stained karyogram for pig #3404 is shown. (TIF) [file pone.0036658.s001.tif]

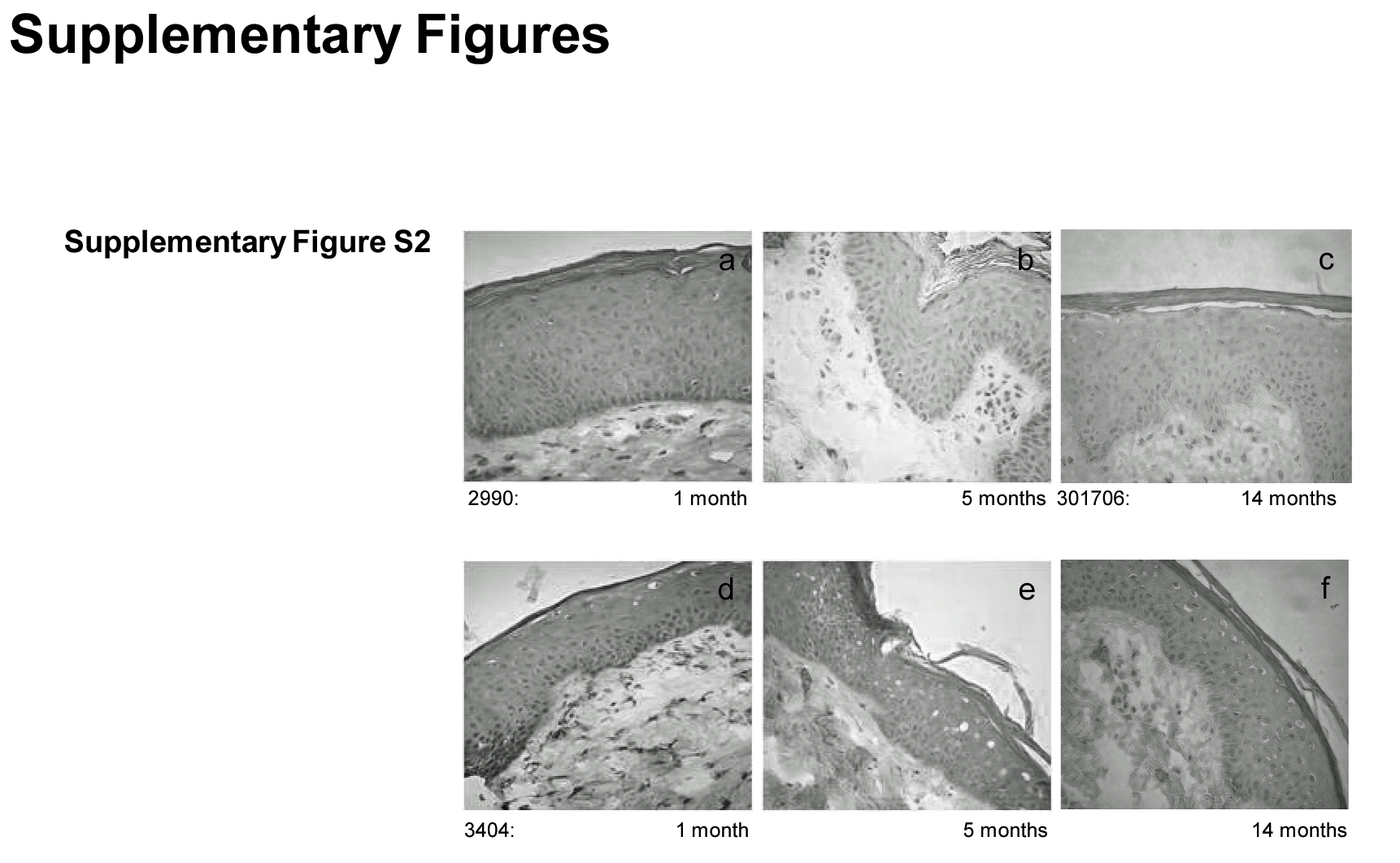

Supplement: Figure S2 — Histological examination of skin sections from hITGB1 transgenic pigs by haematoxylin and eosin staining. Skin biopsies from the six hITGB1-transgenic and control pigs were taken at the age of 1, 5 and 14 months. The biopsies were embedded in OCT, snap frozen in liquid nitrogen, sectioned into 6 µm slices and H&E-stained. No change in skin morphology could be detected in any of the six hITGB1-transgenic pigs over the period of 14 months. Representative pictures are shown for control #2990 (a–b), control #301706 (c) and hITGB1-transgenic pig #3404 (c–e). (TIF) [file pone.0036658.s002.tif]

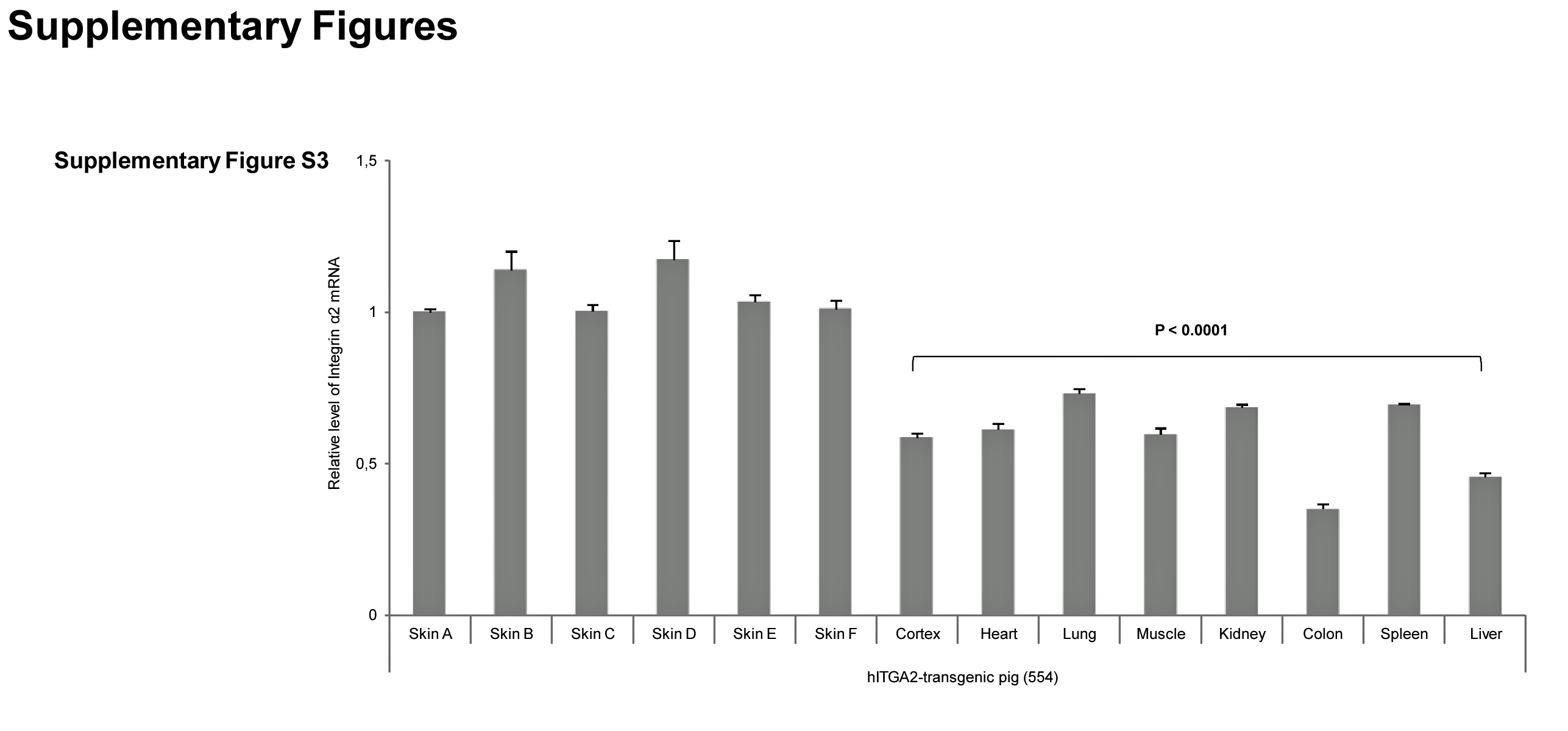

Supplement: Figure S3 — Quantification of hITGA2 mRNA by qRT-PCR in tissues from hITGA2-transgenic pig #554. Tissue biopsies from the sacrificed pig #554 and an age-related wildtype pig were grinded after which total RNA was extracted and employed for hITGA2-directed qRT-PCR, normalized to endogenous β-actin mRNA levels. No hITGA2 was detected in any of the samples from the non-transgenic control pig. Obtained values in pig #554 are shown relative to the level detected in one of the skin samples (skin A) which is set to 1. A statistically significant (p<0.0001) expression was seen in all pig #554 skin samples relative to internal tissues. Skin samples A–F were taken from different locations on pig #554. Data are presented as mean values ± standard deviations. (TIF) [file pone.0036658.s003.tif]

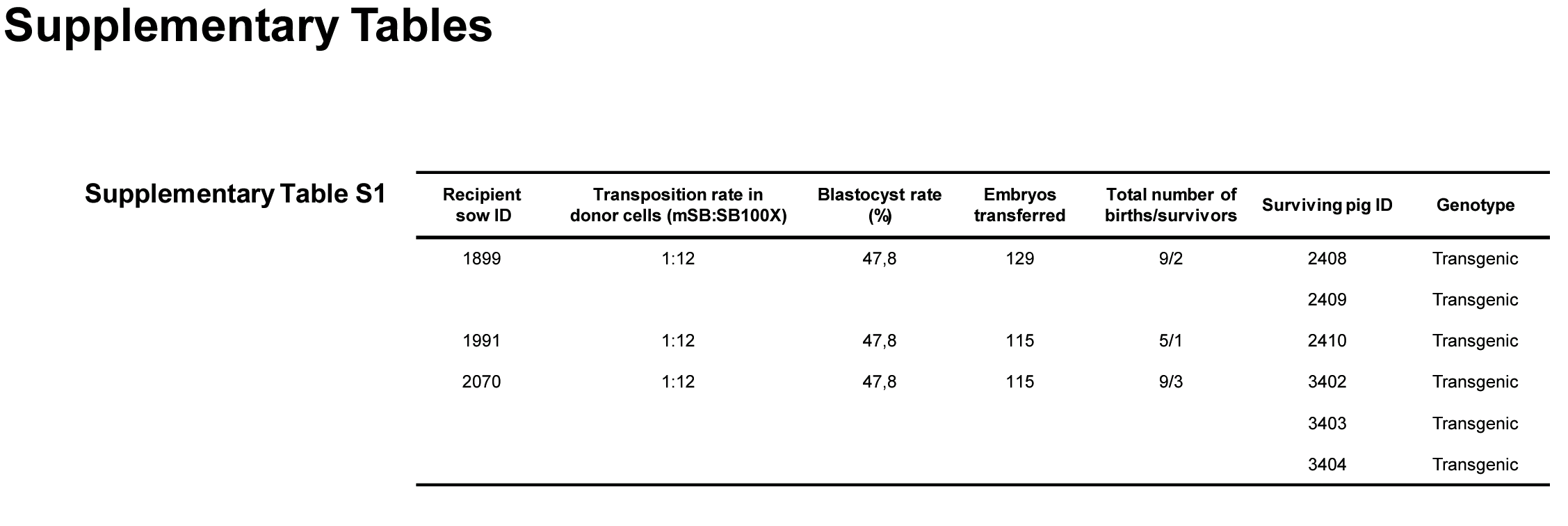

Supplement: Table S1 — Summary of cloning efficiencies obtained with hITGB1-transgenic fibroblasts. (TIF) [file pone.0036658.s005.tif]

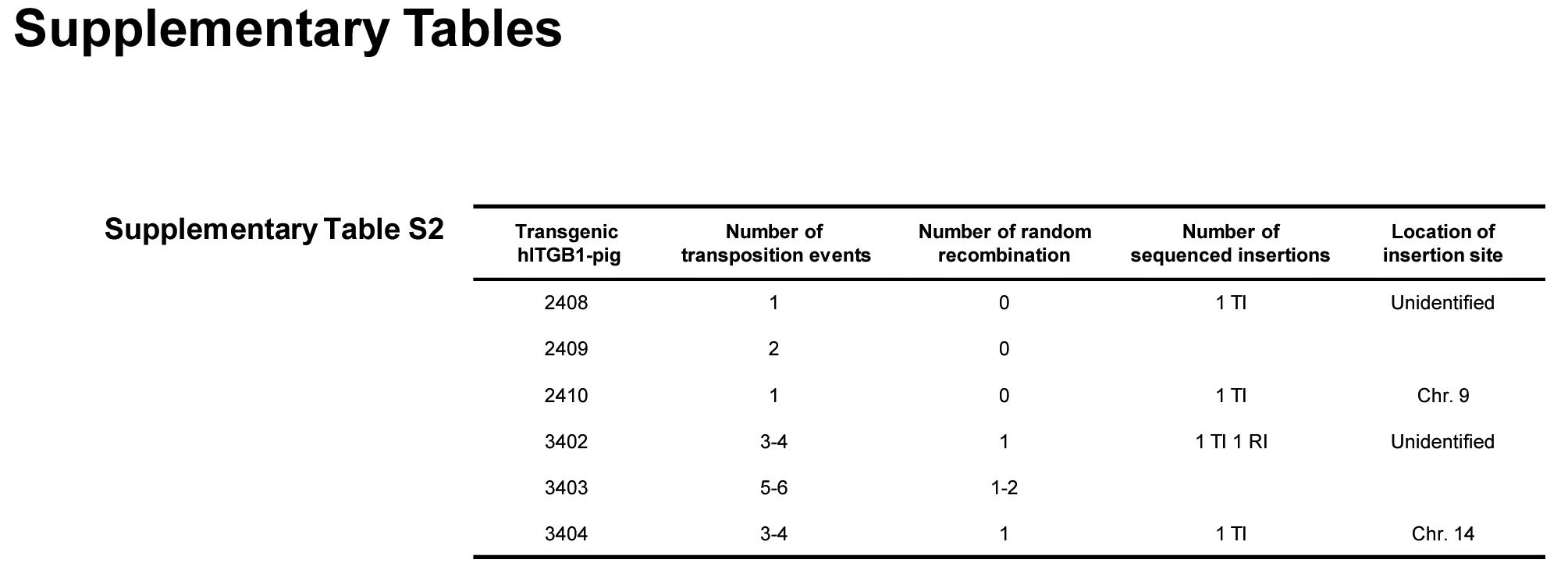

Supplement: Table S2 — Summary of genotyping results obtained in hITGB1-transgenic pigs. (TIF) [file pone.0036658.s006.tif]

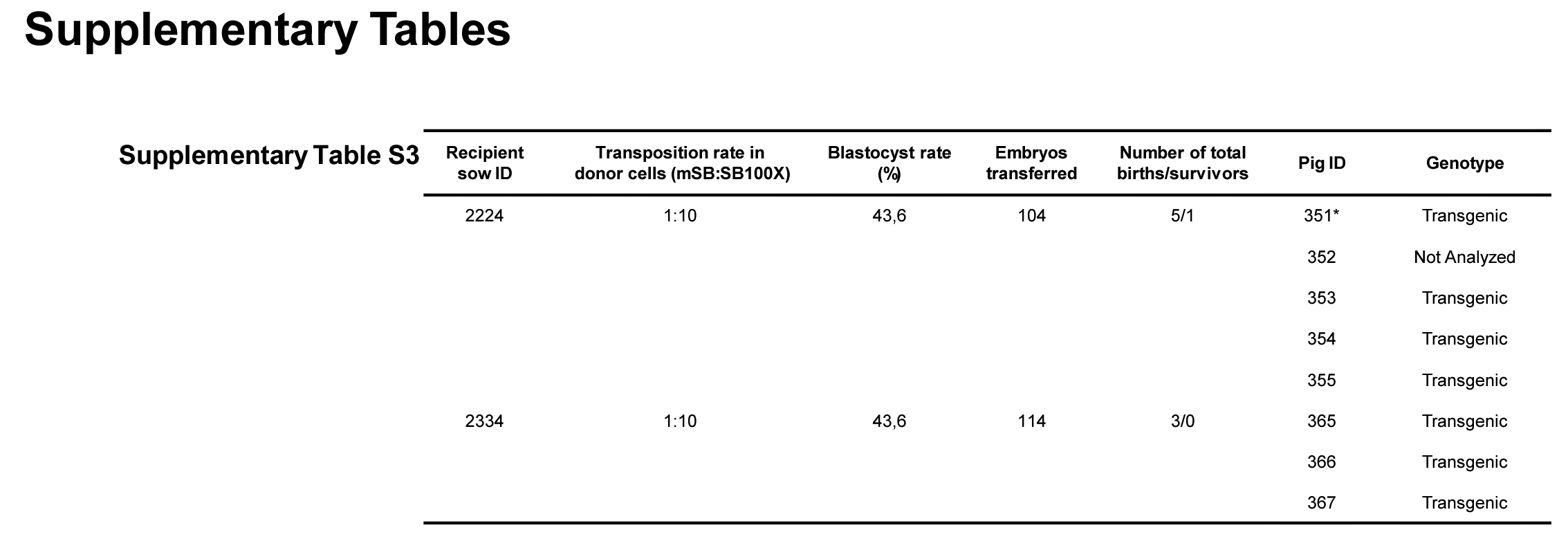

Supplement: Table S3 — Summary of cloning efficiencies obtained with hITGA2-transgenic fibroblasts. The only surviving pig is indicated with an asterisk (*). (TIF) [file pone.0036658.s007.tif]

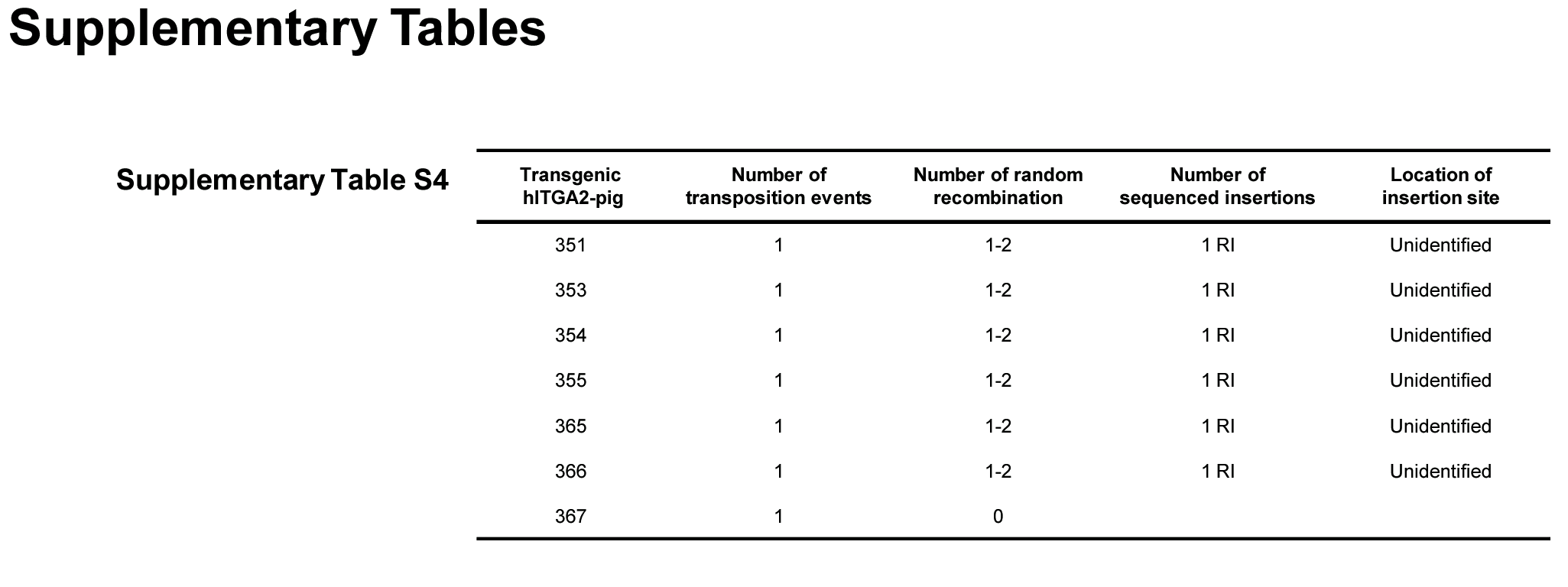

Supplement: Table S4 — Summary of genotyping results obtained in hITGA2-transgenic pigs. (TIF) [file pone.0036658.s008.tif]

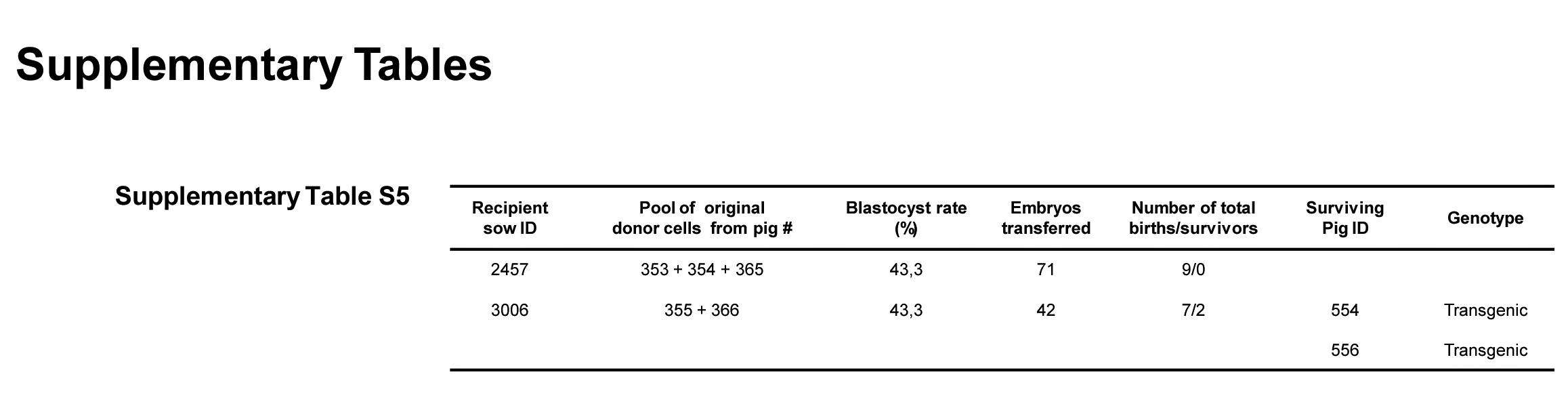

Supplement: Table S5 — Summary of re-cloning efficiencies obtained with original hITGA2-transgenic fibroblasts. (TIF) [file pone.0036658.s009.tif]
