# Supplementary material for: Development of Transgenic Cloned Pig Models of Skin Inflammation by DNA Transposon-Directed Ectopic Expression of Human β1 and α2 Integrin
Source: PLoS One. 2012 May 10;7(5):e36658. doi: 10.1371/journal.pone.0036658 (PMC3349713; doi:10.1371/journal.pone.0036658)
Supplement: Materials and Methods S1 — Transposition assays and generation of donor cells for HMC, H&E staining, PCR and qRT-PCR, Identification of SB insertion sites by LDI-PCR and sequencing, Southern blot analysis, Karyotype analysis, and Western blot analysis. (DOCX) [file pone.0036658.s004.docx]

**SUPPLEMENTARY MATERIALS AND METHODS S1**

**Transposition assays and generation of donor cells for HMC**

For stable transfections using the SB system, NIH3T3 or Göttingen primary fibroblasts were seeded in 6-well plates at a density of 2 × 10^5^ cells/well. On the preceding day the cells were transfected using Fugene 6 (Roche, Basel, Switzerland) according to the manufacturer’s protocol utilizing a 6µL:2µg fugene:total plasmid ratio for Göttingen primary fibroblasts and a 3µL:2µg ratio for NIH3T3 cells. After 24h, the cells were trypsinized and transferred to a P10 dish (Greiner Bio-one GmbH, Frickenhause, Germany). After another 24h, the cells were subjected to G418 (Invitrogen Ltd, Paisley PA4 9RF, UK) selection (1 mg/mL) for 14 days. Subsequently, the resistant colonies were either stained with methylene blue (Sigma-Aldrich, St. Louis, MO, USA) for evaluation of transposition efficiency or isolated and pooled for cloning. Pooled transgenic cells for cloning were re-seeded in one well of a 6-well dish and allowed to expand until confluency. At confluency the cells were re-seeded into 4 wells of a 12-well dish at a density of 3 × 10^4^ cells/well and allowed to expand for three days to reach confluency without change of medium.

**H&E staining**

Air-dried, frozen skin sections from the six hITGB1 transgenic pigs, the non-transgenic control pig and from a non-lesional psoriasis skin sample were placed in 0.1% Mayers Hematoxylin (Sigma, St Louis, MO, USA) for 10 min. Subsequently, the slides were first rinsed in copious amounts of cool ddH_2_O for 5 min and hereafter in Scott’s tap water substitute for 2 min. The slides were successively dipped in 0.5% eosin (dissolved in 95% EtOH) ten times and dipped in ddH_2_O until the eosin stopped streaking. The samples were dehydrated in increasing concentrations of EtOH, 10 dips in 50%, 10 dips in 75%, 30 sec. in 95% and 1 min. in 100% EtOH. Finally, the slides were dipped in Xylene several times and mounted with Organo (Sigma, St Louis, MO, USA).

**PCR and qRT-PCR**

PCR was performed on 100 ng DNA purified from primary fibroblasts from α2 or β2 integrin-transgenic pigs and the non-transgenic pig 3854. Primers specific for the neomycin selection marker (500 bp product), the hITGB1 (280 bp product) and hITGA2 (175 bp product) transgenes, and for the SB100X transposase (390 bp product) were used in a standard Taq polymerase (Ampliqon A/S, Skovlunde, Denmark) PCR protocol. Reactions were run for 35 cycles and visualized on a 1.5% agarose ethidium bromide stained gel.

For qRT-PCR, 150-300 ng RNA extracted through a spin-column (RNeasy kit, QIAGEN, Hilden, Germany) from Göttingen primary keratinocytes, primary fibroblasts as well as from skin and internal tissue biopsies was DNase treated (DNA-free, Ambion, TX, USA) and used for standard 20 µL cDNA synthesis (iScript cDNA synthesis kit, Bio-Rad Laboratories, CA, USA). From total cDNA 1:20 to 1:10 was utilized for the qPCR analysis. Q-PCR analyses were performed according to manufacturer’s instructions. The following TaqMan gene expression assays (Applied Biosystems, CA, USA) were deployed; human ITGA2 detection (Hs00985380), porcine c-Fos detection (Ss03390402), porcine EGFR (Ss03393423), porcine psoriasin (Ss03377162), porcine IL-8 (Ss03392437), porcine CXCL10 (Ss03391846), porcine IL-1β (Ss03393804), porcine TNF-α (Ss03391318), porcine PCNA (Ss03377029), porcine CCL27 (Ss03378478), porcine CCL5 (Ss03648939), porcine CCL20 (Ss03391927) and porcine GM-CSF (Ss03394096). The Taqman probe for porcine IL-1A detection was custom made (FAM- tgctgaaggagctgcctgagacaccc-MGB) and used in combination with the sense primer (gcaacttcctgtgactctaagaatctc) and antisense primer (catattgccatgcttttcccagaa). Measurements of human ITGB1 levels were conducted by use of the DyNAmo HS SYBR Green qPCR Kit (Finnzymes, Espoo, Finland) and primers specific for hITGB1. As internal control for human cells, RPLP02 (Hs99999902) expression was measured and for porcine cells, ACTB (Ss03376081) and GAPDH (Ss03375435) levels were measured. All experiments were run on a LightCycler 400 (Roche, Basel, Switzerland).

**Identification of SB insertion sites by LDI-PCR and sequencing**

For identification of SB insertion sites, genomic DNA was Trizol-extracted (TRI Reagent, Sigma-Aldrich, MO, USA) from fibroblasts cultured from ear biopsies from the transgenic animals. Three µg DNA was digested with XhoI and SalI (New England Biolabs, MA, USA), column-purified and ligated in a total volume of 500 µL ligation mix (Invitrogen, Paisley, UK). Subsequently, 2-5 µL column-purified, ligated DNA was employed as template for the first PCR using a sense primer recognizing the right inverted repeat 5’-CTCGAAATGTTTGACCCAAGTT-3’ and an antisense primer specific for a site within the α2 gene (5’-TGCACTGCATAGCCAAACTG-3’) or the β1 gene (5’-TTGGAGCCTCTGGGATTTTC-3’). From the first round of PCR, 2-5 µL was used as template in a nested PCR using the primers 5’-AGCTGAAATGAATCATTCTCTC-3’ and 5’-CCTTGACTGAGCGCTAACAC-3’ (for α2) or 5’-GTTTGAGCAAACACACAGCA-3’ (for β1). All reaction products were separated by gel electrophoresis and discrete bands were excised and column purified (Gel Extraction kit, Omega Bio-tek, GA, USA). Purified DNA was mixed with the sense primer from either the first or the nested PCR and sequenced (MWG Eurofins, Germany). The resulting sequences were blasted against the annotated pig Sscrofa9 assembly (Ensembl).

**Southern blot analysis**

Between 11 and 15 µg of column-purified genomic DNA was digested O/N at 37˚C with the following restriction enzyme combinations SalI + EcoRV or EcoRV + PvuI for hITGA2 detection and with SalI + NheI or EcoRV + PvuI (New England Biolabs, MA, USA) for hITGB1 detection in a total volume of 200 µL. The digested DNA was NaAC-precipitated and resuspended in 20 µL TE. The DNA fragments were electrophoresed on a 0.8% agarose gel O/N at 1 V/cm and transferred to a nylon membrane (Amersham Hybond –N^+^, GE Healthcare, Buckinghamshire, UK) by vacuum suction. During transfer the DNA was nicked, denatured and neutralized in 0.25 M HCl, 1.5M NaCl + 0.5M NaOH and 1.5M NaCl + 1M Tris-HCl pH 7.6, respectively. Subsequently the membrane was washed with 20 x SSC. The DNA was UV cross-linked to the membrane and pre-hybridized with 0.4 mg/mL salmon sperm ssDNA (D1626, Sigma-Aldrich, St. Louis, MO, USA) in 5 x SSPE (0.75 M NaCl, 0.05 M NaH_2_PO_4_, 0.005 M EDTA) 5 x Denhart (0.1% Ficoll 400, 0.1% polyvinylpyrrolidone, 0.1% BSA), 1 % SDS, 50% formamide, 5 % dextran-sulphate for 4 hours at 42˚C. A neomycin-probe of 790 bp was CTP ^32^P-labelled (Prime-It II random primer labeling kit, Stratagene, Cedar Creek, TX, USA) and mixed with salmon sperm ss-DNA (1mg/mL) and hybridization buffer and finally incubated with the membrane at 42 ˚C O/N. The membrane was washed in 2 x SSPE for 2 x 5 min at 26˚ C and in 2 x SSPE/0.5 % SDS for 2 x 15 min at 53˚ C and finally in 0.2 x SSPE/0.5 % SDS for 1 x 5-15 min at 53˚ C. The membrane was air-dried and enclosed in a cassette for exposure of an X-ray film (Konica Minolta Medical & Graphic Inc., Tokyo, Japan).

**Karyotype analysis**

Fibroblasts from hITGB1 transgenic or non-transgenic pigs were seeded in a slideflask (Nunc A/S, Roskilde, Denmark). Subconfluent cultures were incubated with 10µl/mL colcemid for 20 min, washed in PBS, subjected to hypotonic conditions for 15 min with 60 mM KCl and fixed in a 3:1 ethanol/acetic acid mixture. The slides were dried for 45 min at 25˚C and 38% relative humidity. Subsequently, the cells were stained for 4 min with a 1 µg/mL DAPI solution in 2xSSC. The slides were washed in deionized water and dried at room temperature and analyzed using a Leitz fluorescence microscope (Leica Microsystems CMS GmbH, Wetzlar, Germany) and Quips karyotyping software (Yvsis Inc., San Francisco, CA, USA)

**Western blot analysis**

NIH3T3 and primary porcine keratinocyte cell lysates were prepared in a lysis buffer #9803 (Cell Signaling Technology) containing a cocktail of protease inhibitors (Roche, Basel, Switzerland). Keratinocyte cell lysates were prepared in a lysis buffer containing 50mM Tris-HCl pH 7.6, 2.5% SDS, 10% glycerol, 10 mM β-glycerophosphate, 10 mM NaF, 95 µM natriumortovanadate and a cocktail of protease inhibitors (Roche, Basel, Switzerland). Total protein content was determined using a BCA Protein Assay Kit (Pierce). Lysate corresponding to 10 µg of total protein was subjected to PAGE using 3–8% gradient gels (Bio-Rad Laboratories, CA, USA). Proteins were transferred to nitrocellulose membranes using the iBlot transfer system (Invitrogen, Paisley, UK) at 12V for 25 minutes. Human β1 integrin was detected using two different primary anti-human β1 integrin antibodies, secondary antibodies coupled to HRP (DAKO, Rødovre, Denmark), and ECL Western Blotting Detection Reagents (GE Healthcare). Anti-human β1 integrin rabbit polyclonal antibody AB1952 (Millipore, MA, USA) was used at a final 1:1000 dilution and mouse anti-human β1 integrin monoclonal antibody 610467 (BD transduction laboratories, NJ, USA) at a final concentration of 0.1 µg/ml. Actin was detected using a monoclonal anti-actin antibody, A4700 (Sigma-Aldrich, St. Louis, MO, USA) at a final 1:2000 dilution.
